# Expression Quantitative Trait Locus Mapping in Pulmonary Arterial Hypertension

**DOI:** 10.3390/genes11111247

**Published:** 2020-10-22

**Authors:** Anna Ulrich, Pablo Otero-Núñez, John Wharton, Emilia M. Swietlik, Stefan Gräf, Nicholas W. Morrell, Dennis Wang, Allan Lawrie, Martin R. Wilkins, Inga Prokopenko, Christopher J. Rhodes, on behalf of The NIHR BioResource—Rare Diseases Consortium, UK PAH Cohort Study Consortium

**Affiliations:** 1 National Heart and Lung Institute, Hammersmith Campus, Imperial College London, London SW7 2BU, UK; anna.ulrich15@imperial.ac.uk (A.U.); pablo.otero-nunez17@imperial.ac.uk (P.O.-N.); j.wharton@imperial.ac.uk (J.W.); m.wilkins@imperial.ac.uk (M.R.W.); 2 Department of Medicine, University of Cambridge, Cambridge CB2 3AX, UK; es740@cam.ac.uk (E.M.S.); sg550@cam.ac.uk (S.G.); nwm23@cam.ac.uk (N.W.M.); 3 Pulmonary Vascular Disease Unit, Royal Papworth Hospital NHS Foundation Trust, Cambridge CB2 0AY, UK; 4 NIHR BioResource-Rare Diseases, Cambridge, CB2 0QQ, UK; 5 Department of Haematology, University of Cambridge, Cambridge CB2 3AX, UK; 6 Sheffield Institute for Translational Neuroscience, University of Sheffield, Sheffield S10 2TN, UK; dennis.wang@sheffield.ac.uk; 7 Sheffield Bioinformatics Core, University of Sheffield, Sheffield S10 2TN, UK; 8 Department of Infection, Immunity & Cardiovascular Disease, University of Sheffield, Sheffield S10 2TN, UK; a.lawrie@sheffield.ac.uk; 9 Department of Clinical and Experimental Medicine, University of Surrey, Guildford GU2 7XH, UK; i.prokopenko@surrey.ac.uk; 10 Department of Metabolism, Digestion and Reproduction, Imperial College London, London SW7 2BU, UK

**Keywords:** expression quantitative trait locus, eQTL, pulmonary arterial hypertension, blood, genetics

## Abstract

Expression quantitative trait loci (eQTL) can provide a link between disease susceptibility variants discovered by genetic association studies and biology. To date, eQTL mapping studies have been primarily conducted in healthy individuals from population-based cohorts. Genetic effects have been known to be context-specific and vary with changing environmental stimuli. We conducted a transcriptome- and genome-wide eQTL mapping study in a cohort of patients with idiopathic or heritable pulmonary arterial hypertension (PAH) using RNA sequencing (RNAseq) data from whole blood. We sought confirmation from three published population-based eQTL studies, including the GTEx Project, and followed up potentially novel eQTL not observed in the general population. In total, we identified 2314 eQTL of which 90% were cis-acting and 75% were confirmed by at least one of the published studies. While we observed a higher GWAS trait colocalization rate among confirmed eQTL, colocalisation rate of novel eQTL reported for lung-related phenotypes was twice as high as that of confirmed eQTL. Functional enrichment analysis of genes with novel eQTL in PAH highlighted immune-related processes, a suspected contributor to PAH. These potentially novel eQTL specific to or active in PAH could be useful in understanding genetic risk factors for other diseases that share common mechanisms with PAH.

## 1. Introduction

The relationship between genomic variability, disease risk and endophenotypes has been a focus of many research groups with access to suitable disease cohorts. This has been aided by the increasing affordability of genotyping and sequencing methodologies. Interpretation of the mechanisms behind the effects of genetic variants discovered to associate with phenotypes poses a challenge as many are located in the non-coding space of the genome [1]. Non-coding variants exert no direct effect on protein structure, making the biological link to the disease or phenotype more difficult to discern. Variants in the non-coding space may instead exert their effect by influencing gene expression [1]. Characterising the genomic control of gene expression can offer a handle on understanding the role of disease-associated variants by linking them to disrupted pathways [2]. Variants associated with the expression of a gene, commonly called expression quantitative trait loci (eQTL), have been described, primarily in population-based studies [3,4,5].

Differential gene expression analyses comparing cases and controls may be useful for diagnostic and/or prognostic purposes or in identifying genes, which may have a causal role in developing the disease [6,7,8]. In the case of the latter, distinguishing between differentially expressed genes that are secondary to the disease and those that contribute to disease development is a significant challenge. If the frequency of one or several eQTL for a differentially expressed gene is also different between cases and controls, a causal association may be established, given the assumptions of causal inference [9] are met. In brief, for a causal relationship to be established between a gene product proxied by an eQTL and a disease, the eQTL has to be independent of the confounders of the association between the gene and the disease, and it has to have no direct effect on the disease via a pathway that does not involve the gene being instrumented.

Currently, one of the main limiting factors for causal inference analysis using genomic data is the number of known eQTL. Certain eQTL effects, which go undetected in healthy cohorts commonly used for eQTL mapping, may be unmasked by disease and development. The diagnostic value of gene expression profiles, i.e., that they can be used to distinguish affected individuals from non-affected ones, underlines the uniqueness of the set of genes expressed in a disease state [6,7]. Differential gene expression in diverse states (carcinogenesis, inflammation, etc.) or at diverse stages (such as stages of embryonic development) becomes evident when comparing the gene correlation matrices of a biological system under multiple different conditions. Such techniques are called ‘differential co-expression networks’ [10] and have been used successfully to identify genes and gene sets that are important in the state or stage under investigation in a network of genes [11,12]. By principle, functionally involved nodes or modules rewire more frequently than uninvolved ones in states they create and/or maintain and, therefore, can be identified [13].

With the exception of housekeeping genes, the majority of genes in the human transcriptome are tissue-specific. Therefore, tissue selection for gene expression studies of disease is of importance. The lung is the most relevant tissue in the pathogenesis of pulmonary arterial hypertension (PAH), a disease in which pulmonary vascular remodelling drives right ventricular failure. Whole blood also has relevance to PAH and might capture more than just systemic effects given the immune component of this disease. The role of inflammation in PAH has been extensively studied, based on the observation that inflammatory cells infiltrate the remodeled vascular wall [14]. Since PAH can be a complication of many inflammatory diseases, including connective tissue disease, thyroiditis, scleroderma, systemic lupus erythematosus, human immunodeficiency virus infection and schistosomiasis, it might be reasonable to suspect that inflammatory processes, even in the absence of a diagnosed comorbidity, contribute to disease development and maintenance. Another advantage of using whole blood instead of lung tissue is the non-invasiveness of sampling and availability. Lung samples are often only obtainable from the explanted organ after lung transplantation or from post-mortem tissues. Appropriate control samples for differential gene expression analyses are equally hard to come by.

This study aimed to characterise the genetic variability of transcriptome-wide gene expression in whole blood from 276 consecutively sampled patients with PAH and defined potentially novel eQTL active in this disease state. These potentially disease-specific eQTL could be useful in elucidating known genotype-phenotype associations or causal analyses.

## 2. Materials and Methods

### 2.1. Study Participants and Sample Processing

A total of 276 patients with idiopathic, heritable or drug-induced PAH diagnosed following international guidelines [15] were recruited from expert centres across the UK with whole-genome sequence conducted as part of the UK National Institute for Health Research BioResource (NIHRBR) study [16,17]. Transcriptome profiling through RNAseq was completed as part of the PAH cohort study [18]. Demographic characteristics and white blood cell counts of the individuals in this study are shown in Supplementary Table S1.

### 2.2. Gene Expression Data

RNA sequencing and transcript abundance estimation procedures are described in the Supplementary Material. White blood cell composition was quantified using quanTIseq, a novel deconvolution algorithm [19]. Predicted white blood cell fractions from quanTIseq correlated with clinical white cell fractions available in a subset of PAH patients (Spearman correlation = 0.44–0.73). Well-detected transcripts with a minimum of two reads in at least 95% of samples (*n* = 26,050) were taken forward to the eQTL analyses.

### 2.3. Genetic Data

Whole-genome sequence Hg19-aligned data were available from the NIHRBR study described in detail elsewhere [16]. Genetic variants with a minimum minor allele frequency of 5% were extracted from the NIHRBR variant call format (VCF) files and further filtered for variants called in at least 95% of the samples and had a *p*-value greater than 10^−5^ for Hardy Weinberg equilibrium, leaving a total of 7,362,566 variants for eQTL mapping. Software tools used in the eQTL mapping pipeline included PLINKv1.90 [20] for data extraction and computing multidimensional scaling components, QCTOOLv2.0 [21], for variant filtering, and QUICKTESTv1.1 [22] for association testing.

### 2.4. eQTL Effect Estimation

Raw gene count data were first variance stabilised to achieve homoskedasticity and then quantile normalised to the median distribution. The sample means were then centered to zero, and the sample variance was linearly scaled, such that each sample had a standard deviation of one. A total of 7,362,566 markers were tested for their effects on the expression of 26,050 well-detected genes. Variants within 1 megabase (Mb) on either side of the gene’s transcription start site (TSS) were regarded cis-acting or *cis*-eQTL while all other markers farther on the same chromosome or on different chromosomes were regarded *trans*-eQTL. Each well-detected transcript was modelled as a function of genotype (coded 0, 1 or 2 reflecting the number of alternate alleles) in a linear regression framework while adjusting for the following covariates: Sex, first four components from multidimensional scaling and white blood cell fractions (Supplementary Table S1). The genome-wide results were subsequently pruned using the ‘clump’ command in PLINK 1.90 (parameters: —clump-p1 1.9 × 10^−12^; —clump-r2 0.01; —clump-kb 1000) to pick the lead variant with the lowest *p*-value in a block of variants in linkage disequilibrium, and thus obtain independent effects. Genetic variants that reached the Bonferroni-adjusted significance threshold of 1.9 × 10^−12^ given by dividing the genome-wide significance threshold (*p* ≤ 5 × 10^−8^) by the number of transcripts tested (*n* = 26,050) were considered eQTL in this study.

### 2.5. Confirmation Rate

The confirmation rate of eQTL in this study was calculated in the 2 largest published eQTL studies to date and the Genotype-Tissue Expression (GTEx) Project [3,4,23] (Table 1) to assess the extent to which eQTL in PAH overlapped with eQTL described in healthy populations. These studies were selected for having measured gene expression in the same tissue as our study, in addition to being the largest eQTL mapping studies to date. No eQTL study other than the GTEx Project with available genome-wide results applied RNAseq in whole blood at the time this study was conducted. Confirmation rate was defined as the number of PAH eQTL confirmed by the published study, divided by the total number of PAH eQTL tested by the published study and multiplied by 100 to give a percent value. The matching of gene transcripts and genetic variants between our study and the published studies as well as similarities and differences between the designs of this study and the 3 published studies are described in Table 1 and in the Supplementary Material.

### 2.6. Overlap with Variants from the GWAS Catalog

An important aim of this study was to identify eQTL in PAH with effects that had not been observed in population-based studies and to assess the relevance of these PAH-specific eQTL to related phenotypes and diseases. The NHGRI-EBI GWAS Catalog of published genome-wide association studies (GWAS) [24] provided a curated database of genetic markers associated with a wide range of traits and diseases. All variant-phenotype pairs below an association *p*-value threshold of 9 × 10^−6^ were downloaded from the GWAS Catalog website on 5 March 2020. The GWAS Catalog at the time contained 113,510 unique variants (including variants in linkage disequilibrium [LD]) reported as lead variants for 4314 phenotypes. The list of GWAS variants were intersected with each PAH eQTL (including the lead eQTL and variants in LD with the lead eQTL [*r*^2^ ≥40%]). The difference between the proportions of novel eQTL and previously reported eQTL overlapping lung-related phenotypes and diseases curated from the full GWAS Catalog phenotype list was tested using the 2-sample equality of proportions test.

### 2.7. Functional Enrichment Analysis of Genes with Novel eQTL

Functional enrichment analysis of genes with novel eQTL in PAH using the WEB-based GEne SeT AnaLysis Toolkit (WebGestalt) [25] was conducted to determine if genes with novel eQTL were predominantly from pathways with relevance to the pathobiology of PAH. A form of pathway analysis called over-representation analysis (ORA) [26] was run using the Kyoto Encyclopedia of Genes and Genomes (KEGG) pathways [27] database to group these genes into a smaller number of gene-sets relating to biological processes. We queried the list of genes with novel eQTL whilst all the tested genes also present on the expression array of at least one of the published eQTL studies used for the confirmation process constituted the list of background genes. Pathways below a false-discovery rate (FDR)-corrected significance threshold of 0.05 were considered significant.

## 3. Results

**Figure 1:**
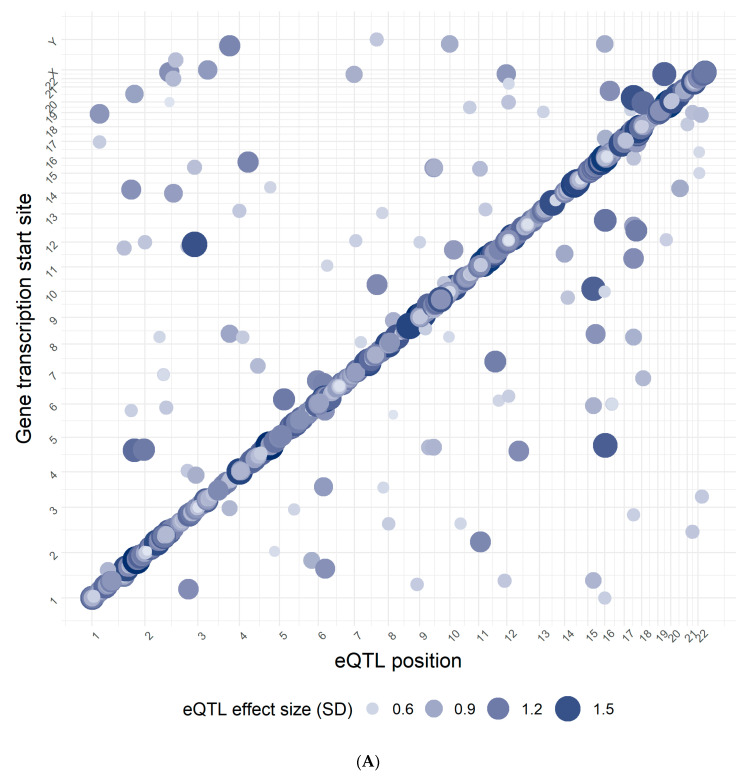

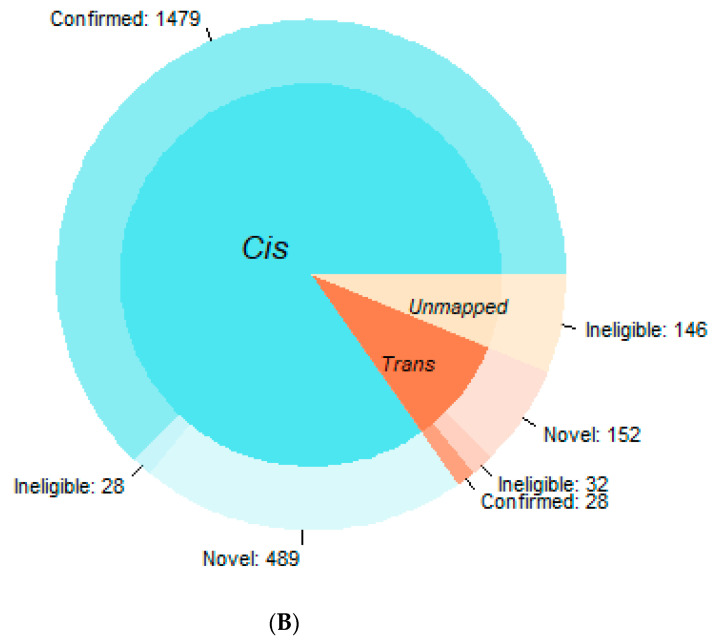
(**A**) Results of the pulmonary arterial hypertension (PAH) Cohort eQTL mapping. Genomic eQTL location (*x*-axis) plotted against the transcription start site of the gene associated with the eQTL. Bubble size and color are proportional to the effect size of the eQTL-gene association. (**B**) Pie chart showing the proportions of *cis*-, *trans*- and unmapped eQTL identified in the PAH Cohort eQTL mapping. Counts of eQTL within each category are shown next to the category name. An eQTL was considered ‘novel’ if it was eligible to be confirmed in at least one of the previously published studies and was not reported as an eQTL previously. Those eQTL that reached the study-specific significance threshold in at least one of the published studies were considered ‘confirmed’. Unmapped transcripts not yet annotated in the Ensembl database could not be confirmed. Ineligible eQTL were not tested by any of the three studies used for confirmation.

We observed 2354 eQTL in total with the majority (90%) acting in cis (Figure 1A), accounting for 9% of all genes tested in this transcriptome- and genome-wide eQTL mapping analysis in PAH whole blood samples. Of these eQTL, 146 were associated with unmapped transcripts not yet annotated in the Ensembl database (Figure 1B). Lead eQTL ranked by the percent of variance explained (R^2^) in gene expression are presented in the Supplementary Table S2. The proportion of variation explained (R^2^) in gene expression levels was generally lower for *trans*-eQTL than for *cis*-eQTL (median_cis-eQTL_ = 23.1%, IQR_cis-eQTL_ = 12.5%; median_trans-eQTL_ = 21.5%, IQR_trans-eQTL_ = 11.5%; t_(df=1332)_ = 4.5, *p*-value = 8.3 × 10^−6^).

### 3.1. Confirmation Rate

Out of the 1986 unique genes with *cis*-eQTL, 1509 (76%) could be mapped to the Illumina HT12v3 array used by Westra et al. and 2134 (95%) to the Affymetrix Human Exon chip used by Joehanes et al., respectively. Results from the GTEx Portal were obtained for 73% of eQTL in this study. Twenty-eight percent of *cis*-eQTL were confirmed by Westra et al., 31% by Joehanes et al. and 90% of tested *cis*-eQTL confirmed in GTEx. Overall, 75% of *cis*-eQTL were confirmed in at least one of the published studies (Figure 1). The overall confirmation rate for *trans*-eQTL reached 16%, with all but one being confirmed by GTEx alone. Joehanes et al. confirmed only one *trans*-eQTL for *JAM3* (chr1:248039451 [C/T]).

### 3.2. Overlap with Variants from the GWAS Catalog

**Figure 2:**
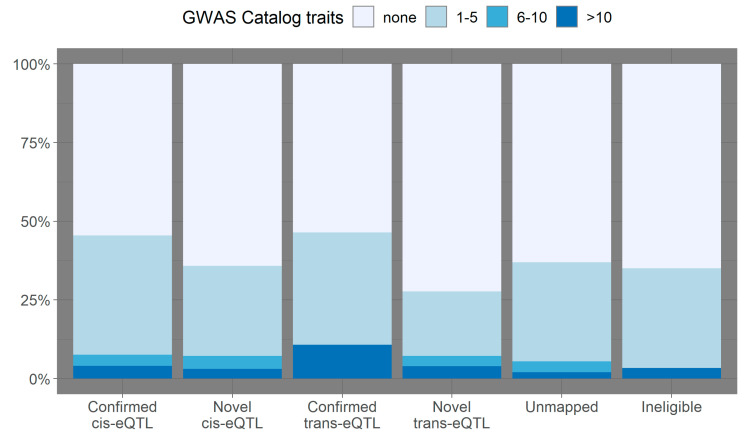
GWAS trait-associations reported for eQTL in the PAH Cohort. Percentage of binned numbers of overlapping GWAS traits (*y*-axis) per eQTL in each eQTL category (*x*-axis). Reported genotype-trait associations were downloaded from the NHGRI-EBI Catalog of published genome-wide association studies.

In order to assess the relevance of eQTL identified in this study to a wide range of phenotypes and diseases, the 2173 unique eQTL were intersected with the database of published genotype-phenotype associations from the GWAS Catalog. In total, 929 eQTL were reported for at least one trait previously (median_N GWAS traits/eQTL_ = 2; IQR_N GWAS traits/eQTL_ = 3). Ninety-eight (11%) of the 929 eQTL had at least 10 or more unique GWAS phenotypes associated with them. Figure 2 shows the number of overlapping GWAS phenotypes per eQTL in the confirmed and novel eQTL categories. The proportion of *cis*-acting eQTL overlapping with at least one published GWAS association in the confirmed subset was 47%, while in the novel subset, it was 37%. Among *trans*-eQTL, the proportion of loci overlapping at least one GWAS trait was 50% in the confirmed and 27% in the novel group. The higher proportion of overlapping loci in the confirmed eQTL groups was significant for both *cis*- and *trans*-acting eQTL (two-sample equality of proportions test *cis*-eQTL: χ^2^_(df=1)_ = 13.9; 95% CI = 0.05–0.15; *p*-value = 0.0002. *trans*-eQTL: χ^2^_(df=1)_ = 4.2; 95% CI = −0.003–0.45; *p*-value = 0.04). Interestingly, the proportion of novel eQTL previously reported for lung-related phenotypes (11%) was twice as high as the proportion of confirmed eQTL associated with lung-related phenotypes (5.5%) (χ^2^_(df=1)_ = 6.9; 95% CI = 0.006–0.1; *p*-value = 0.009). Lung-related phenotypes reported for the novel eQTL included chronic obstructive pulmonary disease (COPD), lung function (forced vital capacity, forced expiratory volume), low versus high forced expiratory volume, interstitial lung disease, emphysema, lung cancer and lung adenocarcinoma. The full list of 63 QTL overlapping lung-related phenotypes in the GWAS Catalog can be found in Supplementary Table S3.

### 3.3. Functional Enrichment Analysis of Genes with Novel eQTL

We assessed the list of 606 genes with novel eQTL in the PAH Cohort for their involvement in certain biological processes or pathways in the KEGG knowledgebase. Five pathways were found to be significantly (FDR <0.05) enriched, with the pathways taste transduction, graft-versus-host disease and autoimmune thyroid disease being the three most significant (Table 2). Supplementary Table S4 lists the overlapping genes driving the association of these five pathways and our results with the corresponding eQTL in PAH, including several human leukocyte antigen (HLA) genes. Furthermore, all genes overlapping with type I diabetes and allograft rejection pathways come from the HLA class. Genes encoded by the more than 200 HLA genes in the highly polymorphic HLA region play an important role in antigen processing and presentation. In total, 20 genes with eQTL in this study came from the HLA classes I and II (*n* = 18) and from the HLA class IB (*n* = 2) genes. Half of these eQTL-HLA gene associations were found to be novel. Out of the 5 *trans*- and 17 *cis*-eQTL, 3 *trans*- and 8 *cis*-eQTL were confirmed by published studies [3,4,23].

## 4. Discussion

Gene expression serves as an intermediate phenotype between genetic variants and associated phenotypes, such as clinical diagnoses, accepted biomarkers and anthropometric and behavioural traits. Previous eQTL studies provided support for the idea that genetic effects on gene expression have phenotypic consequences and that eQTL aid in the biological interpretation of associated genetic markers in disease [3,4,5]. However, eQTL effects are not only dependent on the tissue or cell type under investigation [23,28] but also on the environment or context [5,29,30,31] in which gene expression is measured. This implies that a more comprehensive eQTL map could be constructed by extending mapping efforts beyond population-based studies. We ran a transcriptome-wide eQTL analysis in a cohort of 276 PAH patients to characterise the genetic control of gene expression variability in this condition, uncovering potentially novel eQTL not detected in healthy populations. The resulting 2354 significant eQTL were intersected with the results of two previously published population-based eQTL studies and the results of the GTEx Project. In this study, 25% of *cis*-eQTL and 84% of *trans*-eQTL were not found in any of the three studies used for confirmation. Novel and confirmed eQTL were investigated for their relevance to a wide range of diseases, and results focused on lung-related phenotypes from the NHGRI-EBI Catalog of published genome-wide association studies (GWAS Catalog).

Nearly half (43%) of all eQTL identified in this study colocalised with at least one trait or disease in the GWAS Catalog. Even though the proportion of colocalising eQTL was higher for confirmed *cis*- and *trans*-eQTL than for novel eQTL, the proportion of eQTL associated with lung-related phenotypes was twice as high among novel eQTL than confirmed eQTL previously detected in population-based studies. This indicates that these novel eQTL identified in blood samples of PAH patients might be highly informative for pulmonary diseases such as COPD, interstitial lung disease and emphysema. The higher proportion of GWAS-trait associated eQTL in the confirmed subset might be explained by the lack of replication of novel eQTL by an independent PAH cohort; therefore, it is expected that a proportion of these novel association signals is spurious.

As an example for the overlap between lung-related GWAS traits and eQTL in PAH, the novel *GTF2IRD2B cis*-eQTL rs13238996 (Supplementary Table S3) overlaps with multiple phenotypes including COPD [32], lung function [33], cardiovascular disease [33] and diastolic blood pressure [34]. The Deletion of *GTF2IRD2B* leads to a rare congenital disease called Williams-Beuren syndrome, which frequently presents with supravalvular aortic stenosis (SVAS; OMIM 185500), a congenital heart defect characterised by the narrowing of the aorta, pulmonary and coronary arteries and other blood vessels [35].

Pathway analysis of genes associated with novel eQTL identified five biological processes, including four immune-related phenotypes enriched for this list of genes from the KEGG database. Six genes from the graft-versus-host disease and allograft rejection pathways overlap with the list of genes with novel eQTL, five of which belong to the Human Leukocyte Antigen (HLA) class. The immune-related pathways enriched for genes with novel eQTL in this study demonstrate that novel eQTL could be identified in disease populations since their gene expression profiles differ from the profiles of healthy individuals. The complex pathophysiological mechanisms behind PAH involve multiple driving factors of which immune dysfunction and inflammation are suspected to be among the major contributors (Rabinovitch et al., 2014). The importance of antigen-presenting and recognition in PAH is underlined by the most significant genetic variant discovered in the PAH GWAS in the *HLA-DPA1/DPB1* locus encoding class II major histocompatibility complex (MHC) molecules [16], which associated with three *HLA*-*DPB1* alleles, all containing a glutamic acid at amino acid residue 69 (Glu⁶⁹). The *HLA-DPA1/DPB1* PAH susceptibility locus emphasises the role of immune dysregulation in PAH development [36,37] and warrants further investigation.

Overall, the confirmation rate of eQTL in this study was comparable to that seen in published studies [3,4]. A third of *cis*-eQTL confirmed in the two population-based studies by Westra et al. and Joehanes et al., while 90% of *cis*-eQTL were confirmed by the GTEx Project. Fifteen percent of *trans*-eQTL confirmed in either the GTEx project or the study of Joehanes et al. *Trans*-eQTL validation rates are generally much lower (under 10%) than validation rates of *cis*-eQTL [3,4], reflecting the higher average effect size of *cis*-eQTL and a stronger tissue-specificity of *trans*-eQTL effects compared to *cis*-eQTL, which may render *trans*-eQTL more susceptible to confounding by differing experimental conditions and environmental factors. However, the confirmation rate reported by this study and other studies possibly underestimate the true extent of eQTL overlap between studies since the list of variants and genes that passed quality control and were tested by any one study are usually not made available. We observed a higher confirmation rate with the GTEx Project that also used RNAseq for assaying gene expression. Similarly, Joehanes et al. reported higher validation rates between two array-based studies than between array-based and RNAseq-based studies. This may be related to platform differences, for example, hybridisation in arrays is less sensitive than high-depth sequencing and potentially affected by differing background signals in various tissues. Differences between the study populations being compared can also give rise to imperfect validation and also to novel discoveries, as all genetic effects depend on both the genetic (epistasis) and environmental context of the population they were estimated in and, therefore, do not necessarily apply to another population or the same population at a different time. A more accurate way of identifying novel eQTL in this study would have been to contrast PAH eQTL effects with eQTL effects estimated in a control population assayed on the same platforms and in one batch with the PAH samples to reduce the variability due to technical factors. In addition, novel eQTL are yet to be replicated in an independent population of PAH patients to identify true positives.

The effects of eQTL can vary by the tissue and even cell type under investigation [28,38] and may be modified by external and environmental factors [29,30]. Previous studies have successfully identified ‘response’ eQTL that are associated with gene expression levels in cells under one of two experimental conditions, but not both. For example, in the study of Barreiro, nearly 200 eQTL were identified in primary dendritic cells from 65 individuals with effects specific to either the Mycobacterium tuberculosis-infected cells or the uninfected ones [29]. These response eQTL were argued to constitute natural regulatory variation that likely affect host-Mycobacterium tuberculosis interaction and account for interindividual variation in the immune response and susceptibility of tuberculosis. The authors of the study found when integrating their data with genome-wide genetic susceptibility of pulmonary tuberculosis that these response eQTL were more likely to be associated with the disease, uncovering potential susceptibility genes in pulmonary tuberculosis. Another study by Fairfax et al. [30] investigated the effect of innate immune stimuli on eQTL effects by exposing primary CD14+ cells from 432 European volunteers to the inflammatory cytokine interferon-γ or the endotoxin lipopolysaccharide. Inflammatory stimulation revealed hundreds of eQTL specific to either stimulus, which were enriched for disease-risk loci. In this study, the proportion of eQTL also associated with lung diseases or lung function was twice as high in the novel eQTL subset than in the confirmed subset, highlighting the importance of genotype-environment interaction in understanding the genetic variation of disease susceptibility characterised by genome-wide association studies.

To date, large-scale eQTL-mapping has been done in healthy individuals from population-based studies (Joehanes et al., 2017, Westra et al., 2013, Zhernakova et al., 2017), providing a valuable knowledge base for understanding associations between genetic variation and various phenotypes. It has been shown that by recapitulating the environmental context relevant to disease, it is possible to decipher the genetic variation of disease susceptibility more extensively (Fairfax et al., 2014, Barreiro et al., 2012, Çalışkan et al., 2015). Genome- and transcriptome-wide eQTL-mapping in this cohort of idiopathic and heritable PAH patients identified hundreds of potentially novel eQTL with twice the proportion of lung disease associated with genetic variants than eQTL confirmed by population-based studies. Apart from pulmonary conditions, these novel eQTL specific to, or active in, PAH could be useful in understanding genetic risk factors for other diseases that share common mechanisms with PAH, such as those with immune dysregulation.

## Figures and Tables

**Table 1:** Characteristics of the PAH Cohort eQTL mapping study and published eQTL studies used for confirmation.

	Westra et al.	Joehanes et al.	GTEx	This Study
Gene expression array	Illumina HumanHT-12 v4.0	Affymetrix HuEx 1.0 ST	RNAseq	RNAseq
Genotyping panel	HapMap2	1000-Genomes	WGS	WGS
MAF threshold	≥5%	≥1%	≥1%	≥5%
*n* variants in analysis	not reported	8,510,936	10,008,325	7,362,566
*cis*-eQTL	≤250 kb from PMP	≤1 Mb from the TSS	≤1 Mb from the TSS	≤1 Mb from the TSS
*trans*-eQTL	≥5 Mb from PMP	>1 Mb from the TSS	>1 Mb from the TSS	>1 Mb from the TSS

Comparison of gene expression- and genetic data and expression quantitative trait locus (eQTL) definitions. MAF = minor allele frequency; PMP = probe midpoint; TSS = transcription start site; RNAseq = RNA sequencing; WGS = whole-genome sequencing.

**Table 2:** Pathways enriched for genes with novel eQTL in the PAH Cohort.

Gene Set	Description	Size	Overlap	Expectation	Enrichment Ratio	*p*-Value	FDR
hsa04742	Taste transduction	48	8	0.87	9.24	1.78 × 10^−6^	5.72 × 10^−4^
hsa05332	Graft-versus-host disease	32	6	0.58	10.40	1.85 × 10^−5^	2.86 × 10^−3^
hsa05320	Autoimmune thyroid disease	34	6	0.61	9.79	2.66 × 10^−5^	2.86 × 10^−3^
hsa05330	Allograft rejection	33	5	0.59	8.40	2.75 × 10^−4^	0.02
hsa04940	Type I diabetes mellitus	36	5	0.65	7.70	4.19 × 10^−4^	0.03

Results of the over-representation analysis using the Kyoto Encyclopedia of Genes and (KEGG) pathway database. Column names: Gene set = searchable identifier of the gene set in KEGG; Size = number of genes participating in the gene set and overlap gives the number of genes in the gene set that overlap with the queried gene list; Expectation = number of genes that are expected to be shared between the queried gene list and the gene set if the queried list was a random sample of the background gene list; Enrichment ratio = fold enrichment of the gene set for the queried gene list; *p*-value = enrichment significance of the Fisher‘s exact test; FDR = false discovery rate adjusted significance.

## Supplementary Materials

The following are available online at https://www.mdpi.com/2073-4425/11/11/1247/s1, Table S1: Patient characteristics and white blood cell fractions in the PAH Cohort study, Table S2: Lead variants for novel eQTL in the PAH Cohort ordered by variance explained in gene expression, Table S3: Phenotypes from the NHGRI-EBI GWAS Catalog of published genome-wide association studies reported for novel and confirmed eQTL, Table S4: Significant KEGG pathways from the enrichment analysis of genes with novel eQTL in the PAH Cohort.
