# Supplementary material for: Expression Quantitative Trait Locus Mapping in Pulmonary Arterial Hypertension
Source: Genes (Basel). 2020 Oct 22;11(11):1247. doi: 10.3390/genes11111247 (PMC7690609; doi:10.3390/genes11111247)
Supplement: Supplementary file 1 [file genes-11-01247-s001.pdf]

Supplementary Appendix

for

Expression quantitative trait locus mapping in pulmonary arterial hypertension

Anna Ulrich *et al.*

RNA sequencing and transcript abundance estimation 2

eQTL validation procedure........ 2

eQTL studies used for validation........ 3

Supplementary Tables 4

RNA sequencing and transcript abundance estimation

Whole blood (3ml) was collected in Tempus™ Blood RNA Tubes, which were stored at -80 °C until required. RNA was extracted using a Maxwell robotic system (Promega). Samples with a 260/230 ratio >1.5 and a 260/280 ratio in the range 1.9-2.1 were further quality checked by Bioanalyser and those achieving a minimum RNA Integrity Number (RIN) of 7 were submitted for sequencing. Globin-Zero Gold rRNA Removal Kits (Illumina Inc, San Diego, CA) were used to remove ribosomal RNA contamination from whole blood RNA samples. 75bp paired-end sequencing on a Hiseq4000 was performed on pooled libraries of ~80 samples.

Fastq files (raw reads from RNAseq) were analysed using Salmon v0.9.1 (Patro et al., 2017) and GENCODE release 28 to produce transcript abundance estimates which were converted to gene expression data using tximport in R (Soneson et al., 2015). Salmon, the first transcriptome-wide quantifier to correct for fragment GC-content bias was used, which substantially improves the accuracy of abundance estimates and the sensitivity of subsequent differential expression analysis (Patro et al., 2017).

eQTL validation procedure

To assess the extent to which expression quantitative trait locus (eQTL) - transcript pairs in PAH overlap with previously reported eQTL-transcript pairs described in healthy populations, we calculated the validation rate of our findings in the two largest published eQTL studies to date and the Genotype-Tissue Expression (GTEx) Project (Westra et al., 2013, Joehanes et al., 2017, Aguet et al., 2019). Validation rate was defined as the number of significant eQTL-transcript pairs in this study confirmed by the published study divided by the total number of significant eQTL-transcript pairs that were tested by the published study and multiplied by one hundred.

We extracted all eQTL with effects below the study specific significance threshold from the published studies` results for all significant transcripts in this study. Ensembl identifiers were used for the transcripts and genomic coordinates (chromosome and base pair position) on the Genome Reference Consortium Human Build 37 for eQTL when matching my eQTL-transcript pairs to those published by the external studies.

An eQTL-transcript pair was considered confirmed if the lead variant was in linkage disequilibrium ($r^2 \geq 40\%$) with the lead eQTL of the same transcript in the published study. For this purpose, a list of correlated variants was obtained from the European population of the 1000 Genomes Project (Durbin et al., 2010) - using the R package ‘proxysnps’ - for each lead eQTL reported by the studies used for validation. Each eQTL-transcript pair that reached the study-specific significance threshold in at least one of the studies was considered confirmed.

Since the complete list of variants or transcripts that passed study-specific quality control is not usually made available by published studies, we had to make assumptions when determining the total number of my significant eQTL-transcript pairs tested in the external studies. This did not apply to GTEx results where all tested eQTL-transcript pairs could be retrieved.

All transcripts present on the expression array used in the other two studies were assumed to have been tested. Annotation files were downloaded from the manufacturer`s website for the complete list of transcripts present on the expression arrays. Additionally, all eQTL in this study were assumed

to have been available for analysis in the studies used for validation which used genotyping array data imputed to high-density reference panels. We restricted our analyses to common variants with a minimum minor allele frequency of 5% which is at least as high as those of the published eQTL studies used for validation.

eQTL studies used for validation

We selected two of the largest published eQTL studies and the GTEx Project to compare our results to. Westra et al. (Westra et al., 2013) meta-analysed eQTL effects from seven studies totaling 5,311 individuals to identify cis-acting effects genome-wide as well as trans-acting effects of 4,542 variants implicated in diseases and traits from the GWAS Catalog (Buniello et al., 2019) at the time of their study. The other eQTL study used for validation was published by Joehanes et al. (Joehanes et al., 2017) who conducted the largest to-date single cohort transcriptome-wide analysis testing both cis- and trans-acting elements genome-wide in the whole blood samples of 5,257 individuals from the Framingham Heart Study. The GTEx Project aims to create a database of genotype and gene expression correlations in multiple human tissues of consenting donors to aid the scientific community in understanding inherited disease susceptibility. The GTEx website provides a browser (https://gtexportal.org/home/testyourown) for retrieving results for any given variant and transcript pair based on Ensembl identifiers in the tissue of interest.

The current release (V8) of the GTEx Project has 838 post-mortem donor samples with genetic data of which 670 contributed to the eQTL mapping in whole blood (GTExPortal, Aguet et al., 2019). Only tissue samples that passed histological examination were accepted for the project, however tissue exclusions were not made based on cause of death. Demographics and cause of death statistics can be found on the GTEx Portal (https://gtexportal.org/).

Westra et al. meta-analysed seven studies that measured gene expression in peripheral blood on one of Illumina`s whole-genome Expression BeadChips (HT12v3, HT12v4 or H8v2 arrays). Since most of the cohorts used the HT12v3 array, the analyses were restricted to transcripts present on this array (Westra et al., 2013). Joehanes et al. used the Affymetrix Human Exon ST 1.0 array for the whole cohort (Joehanes et al., 2017). The complete list of transcripts was obtained from the manifest files, namely HumanHt-12_V3_0_R3_11283641_A.bgx for Illumina and HuEx-1_0-st-v2.na33.1.hg19.probeset.csv for Affymetrix, available on the manufacturers` websites.

The genotype data from Westra et al. and Joehanes et al. were imputed to the largest haplotype reference panels available at the time of conducting their analyses. Westra et al. mapped cis-eQTL differently from our and the other two studies` approach, using a 250 kilobases (kb) maximum distance from the probe midpoint to demarcate cis effects, while eQTLs with a distance greater than 5 Mb were defined as trans-eQTLs. The validation rate of our trans eQTL was not assessed using Westra`s trans eQTL results as they have only tested a small set of selected variants. Also, cis-eQTL in this study were restricted to the ones not farther than 250 kB from the transcript`s TSS when validating cis-eQTL with Westra`s results.

Supplementary Tables

Supplementary Table 1 Patient characteristics and white blood cell fractions in the PAH Cohort study 5
Supplementary Table 2 Lead variants for novel eQTL in the PAH Cohort ordered by variance explained in gene expression. 6
Supplementary Table 3 Phenotypes from the NHGRI-EBI GWAS Catalog of published genome-wide association studies reported for novel and confirmed eQTL. 21
Supplementary Table 4 Significant KEGG pathways from the enrichment analysis of genes with novel eQTL in the PAH Cohort. 27

Characteristic	Males	Females
**N (%)**	82 (30)	194 (70)
**Age at diagnosis, yr**	45.6 (24.5)	47.7 (22.1)
**Age at sampling, yr**	54.4 (22.8)	52.9 (21.8)
**B cells**	0.028 (0.014)	0.028 (0.016)
**Alternatively activated macrophages**	0.022 (0.008)	0.020 (0.011)
**Neutrophils**	0.265 (0.098)	0.285 (0.100)
**Natural killer cells**	0.020 (0.017)	0.020 (0.020)
**CD8+ T cells**	0.050 (0.057)	0.050 (0.038)
**Regulatory CD4+ T cells**	0.150 (0.128)	0.160 (0.128)
**Other uncharacterized cells**	0.397 (0.117)	0.392 (0.141)

Supplementary Table 1 Patient characteristics and white blood cell fractions in the PAH Cohort study. Age and white blood cell fractions are presented as median (interquartile range).

Supplementary Table 2 Lead variants for novel eQTL in the PAH Cohort ordered by variance explained in gene expression.

Column names: eQTL rs ID = dbSNP (150) variant identifier; eQTL chr:pos = chromosome and genomic position (Ensembl GRCh37 coordinates); ref = reference allele in the human genome reference GRCh37; alt = alternative allele in the human genome reference GRCh37; Ensembl gene ID = Ensembl stable gene identifiers; variant type = dbSNP version 150 variant annotation; R^2 = percent (%) variance explained in gene expression by the genetic variant; Gene type: PC = protein coding, TC- = transcribed, PPG = processed pseudogene, unPPG = unprocessed pseudogene, lncRNA = long non-coding RNA, TR V gene = T-cell receptor variable gene, IG V gene = immunoglobulin variable gene, snRNA = small nuclear RNA, snoRNA = small nucleolar RNA, miRNA = micro RNA.

**eQTL rs ID**	**eQTL chr:pos**	**ref**	**alt**	**Gene symbol**	**Ensembl gene ID**	**cis/trans**	**eQTL P-value**	**R^2**	**Gene type**
rs2295768	6:2954602	C	T	*HTATSF1P2*	ENSG00000271361	cis	7.1x10-83	57.4	PPG
rs2240101	15:31115709	G	C	*RP5.1086D14.5*	ENSG00000259845	cis	4.5x10-79	56.2	unPPG
rs2302559	7:105903904	T	C	*NAMPTL*	ENSG00000229644	trans	6.35x10-78	55.9	PPG
rs745666	17:72744798	G	C	*RP11.452I5.2*	ENSG00000266036	cis	1.9x10-70	53.3	PC
rs7635	11:721570	C	T	*RPLP2*	ENSG00000177600	cis	2.05x10-70	53.3	lncRNA
rs471521	12:10115647	A	T	*CLEC12B*	ENSG00000256660	cis	1.94x10-68	52.5	PC
rs67933574	12:11226237	A	G	*TAS2R43*	ENSG00000255374	cis	8.58x10-65	51.1	PC
rs11079015	17:39975164	C	T	*NT5C3B*	ENSG00000141698	cis	2.28x10-64	51	PC
rs2280370	16:89627460	T	G	*RPL13P12*	ENSG00000215030	trans	1.72x10-62	50.2	TCunPPG
rs9892882	17:18501596	A	G	*USP32P1*	ENSG00000188933	trans	2.03x10-62	50.2	PPG
rs9892882	17:18501596	A	G	*CCDC144A*	ENSG00000170160	trans	3.71x10-62	50.1	PC
rs72785089	16:70005967	A	G	*CLEC18A*	ENSG00000157322	cis	1.14x10-61	49.9	PC
rs10947675	6:37479972	G	A	*RP1.153P14.8*	ENSG00000204110	cis	4.66x10-60	49.2	lncRNA
rs1455979	21:44524006	G	A	*U2AF1*	ENSG00000160201	cis	2.5x10-59	48.9	PC
rs12975591	19:10627814	A	G	*CTC.539A10.4*	ENSG00000267100	cis	1.6x10-58	48.5	lncRNA
rs10790962	11:128385169	G	A	*Sep-03*	ENSG00000100167	trans	4.37x10-57	47.9	PC
rs12225372	11:74604594	A	G	*XRRA1*	ENSG00000166435	cis	4.79x10-56	47.4	PC
rs2732603	17:44367288	G	C	*RP11.197O15.1*	ENSG00000214401	cis	5.07x10-55	46.9	lncRNA
rs79240608	1:149585945	T	C	*NBPF26*	ENSG00000273136	trans	6.4x10-55	46.9	PC
rs2336136	14:74030518	T	A	*ACOT1*	ENSG00000184227	cis	1.12x10-54	46.8	PC
rs2286466	16:2014283	A	G	*RPS2P5*	ENSG00000240342	trans	4.46x10-54	46.5	PPG
rs2656417	17:18475630	T	C	*USP32P2*	ENSG00000233327	cis	7.58x10-54	46.4	TCunPPG
rs55969088	18:9024224	G	T	*NDUFV2P1*	ENSG00000267809	trans	7.55x10-54	46.4	PPG
rs9264607	6:31237605	G	A	*HLA.L*	ENSG00000243753	trans	2.13x10-53	46.2	TCunPPG
rs61744075	8:23288346	C	A	*NKX3.1*	ENSG00000167034	cis	1.92x10-52	45.7	PC
rs17847967	12:8082367	T	C	*SLC2A14*	ENSG00000173262	cis	2.4x10-52	45.6	PC
rs11677238	2:114424974	T	G	*AC002055.4*	ENSG00000184319	trans	4.55x10-52	45.5	TCunPPG
rs73287153	7:12139272	A	G	*VWDE*	ENSG00000146530	cis	7.37x10-52	45.4	PC
rs216257	6:24934664	A	T	*LSAMP*	ENSG00000185565	trans	1x10-50	44.8	PC
rs10489762	1:183570545	G	T	*CTD.2058B24.2*	ENSG00000259048	trans	1.84x10-50	44.7	lncRNA
rs2732603	17:44367288	G	C	*RP11.583F2.1*	ENSG00000264057	trans	2.02x10-50	44.7	TCunPPG
rs111839387	7:65285169	C	T	*PSPH*	ENSG00000146733	trans	5.95x10-50	44.4	PC
rs12447763	16:23753594	G	T	*RP11.151E14.1*	ENSG00000261035	trans	8.42x10-50	44.4	lncRNA
rs9934565	16:87764267	A	T	*RP11.278A23.2*	ENSG00000260671	cis	1.73x10-49	44.2	lncRNA
rs2732617	17:44363632	A	C	*RP11.359G22.2*	ENSG00000230400	trans	4.78x10-49	44	lncRNA

eQTL rs ID	eQTL chr:pos	ref	alt	Gene symbol	Ensembl gene ID	cis/trans	eQTL P-value	R^2	Gene type
rs9273495	6:32628273	G	A	*HLA.DQB2*	ENSG00000232629	cis	1.07x10-48	43.8	PC
rs4976210	5:70307464	T	C	*SERF1A*	ENSG00000172058	cis	1.65x10-48	43.7	PC
rs2732603	17:44367288	G	C	*DND1P1*	ENSG00000264070	cis	2.68x10-48	43.6	PPG
rs145140188	11:77627431	G	C	*INTS4P1*	ENSG00000164669	trans	4.36x10-48	43.5	TCunPPG
rs2294641	1:11721854	C	T	*FBXO44*	ENSG00000132879	cis	1.12x10-46	42.7	PC
rs111164082	17:44047037	T	A	*RP11.798G7.3*	ENSG00000214425	cis	1.8x10-46	42.6	TCunPPG
rs6085421	20:6142259	T	A	*RP11.199O14.1*	ENSG00000229876	cis	2.95x10-46	42.5	lncRNA
rs471521	12:10115647	A	T	*AC091814.2*	ENSG00000225231	cis	8.12x10-46	42.2	lncRNA
rs10409076	19:45057224	G	T	*CEACAM22P*	ENSG00000230666	cis	1.68x10-45	42.1	TCunPPG
rs12912107	15:48520590	G	A	*C10orf111*	ENSG00000176236	trans	2.46x10-45	42	PC
rs150996856	7:76596954	G	A	*CCDC146*	ENSG00000135205	cis	2.44x10-45	42	lncRNA
rs2272550	14:22591988	C	A	*TRAV26.1*	ENSG00000211807	cis	2.02x10-45	42	TR V gene
rs739239	22:50050468	G	A	*RP1.29C18.10*	ENSG00000212939	cis	2.29x10-45	42	lncRNA
rs4774967	15:57855760	G	T	*GCOM1*	ENSG00000137878	cis	1.88x10-44	41.5	PC
rs7207087	17:46917953	C	G	*CALCOCO2*	ENSG00000136436	cis	3.11x10-44	41.4	PC
rs7952689	11:7492630	T	G	*CTD.2516F10.2*	ENSG00000251364	cis	7.99x10-44	41.1	lncRNA
rs62557076	9:69449629	T	C	*ANKRD20A4*	ENSG00000172014	cis	2.74x10-42	40.2	PC
rs67851010	6:32501347	G	A	*HLA.DRB5*	ENSG00000198502	cis	4.55x10-42	40.1	PC
rs3179969	14:88862529	G	A	*SPATA7*	ENSG00000042317	cis	1.43x10-41	39.8	PC
rs236628	7:75023850	G	T	*AC006014.6*	ENSG00000205583	cis	2.03x10-41	39.7	TCunPPG
rs330924	8:8999187	G	C	*RP11.10A14.3*	ENSG00000254340	cis	4.11x10-41	39.5	lncRNA
rs9408297	9:48559	G	T	*WASHC1*	ENSG00000181404	cis	2.19x10-40	39.1	PC
rs12047480	1:198635256	A	G	*WISP2*	ENSG00000064205	trans	5.28x10-40	38.8	PC
rs1141684	16:2014591	C	T	*RP11.364L4.1*	ENSG00000228981	trans	3.44x10-39	38.3	PC
rs9264669	6:31239681	A	T	*HLA.C*	ENSG00000204525	cis	4.11x10-39	38.3	PPG
rs10931780	2:198175297	G	C	*AC013264.2*	ENSG00000231621	cis	9.22x10-39	38.1	lncRNA
rs76909629	2:68258988	T	C	*C1D*	ENSG00000197223	cis	1.81x10-38	37.9	PC
rs10130430	14:37448518	T	C	*SLC25A21*	ENSG00000183032	cis	2.1x10-38	37.8	PC
rs62072802	17:18490410	A	G	*RP11.219A15.1*	ENSG00000266302	trans	2.19x10-38	37.8	PC
rs61809765	1:149660625	A	G	*NBPF8P*	ENSG00000270231	trans	1.54x10-37	37.3	TCunPPG
rs17816376	15:90233553	T	C	*PEX11A*	ENSG00000166821	cis	1.88x10-37	37.2	PC
rs11765305	7:143111112	C	G	*AC092214.10*	ENSG00000229153	cis	2.68x10-37	37.1	PC
rs2714313	6:7331682	G	C	*DSP*	ENSG00000096696	cis	2.58x10-37	37.1	lncRNA
rs11772895	7:143081942	G	C	*TAS2R41*	ENSG00000221855	cis	3.57x10-37	37	PC
rs5761004	22:23978677	G	A	*DRICH1*	ENSG00000189269	cis	5.78x10-37	36.9	PC
rs1451659	11:4439535	T	C	*OR51R1P*	ENSG00000237272	cis	9.34x10-37	36.8	PC
rs7093	17:3566232	C	T	*TAX1BP3*	ENSG00000213977	cis	7.11x10-37	36.8	unitary PG
rs62192251	2:231277825	G	T	*RP11.634B22.4*	ENSG00000254467	trans	1.36x10-36	36.6	lncRNA
rs7225024	17:57780223	C	A	*TMEM148*	ENSG00000179219	trans	2.14x10-36	36.5	PC
rs7575806	2:201632512	G	C	*ORC2*	ENSG00000115942	cis	2.52x10-36	36.5	lncRNA
rs16995310	20:1550334	C	A	*RP4.576H24.4*	ENSG00000260861	cis	2.69x10-36	36.4	PC
rs12415	2:232573292	T	C	*PTMA*	ENSG00000187514	cis	5.05x10-36	36.3	PC
rs13099099	3:132441268	T	C	*NPHP3*	ENSG00000113971	cis	5.1x10-36	36.3	PC
rs2732603	17:44367288	G	C	*MAPK8IP1*	ENSG00000121653	trans	4.15x10-36	36.3	PC

eQTL rs ID	eQTL chr:pos	ref	alt	Gene symbol	Ensembl gene ID	cis/trans	eQTL P-value	R^2	Gene type
rs376215580	1:146385910	A	G	*NBPF12*	ENSG00000268043	trans	1.2x10-35	36	PC
rs1510881	4:144439082	T	C	*FREM3*	ENSG00000183090	cis	2.13x10-35	35.8	PC
rs28408686	17:30846624	C	G	*CDK5R1*	ENSG00000176749	cis	3x10-35	35.7	PC
rs12962555	18:46901634	T	A	*RPL17.C18orf32*	ENSG00000215472	cis	4.32x10-35	35.6	PC
rs72843816	10:131846688	T	C	*RP11.500G10.1*	ENSG00000237489	cis	7.05x10-35	35.5	PC
rs9273841	6:32629582	C	T	*RP11.544M22.1*	ENSG00000198358	trans	1.66x10-34	35.2	lncRNA
rs375407631	9:66470329	A	G	*RP11.262H14.3*	ENSG00000234665	cis	3.48x10-34	35	lncRNA
rs67651814	20:43598154	G	C	*RP11.369C8.1*	ENSG00000258616	trans	3.62x10-34	35	lncRNA
rs11709127	3:20228009	T	C	*AC099057.8*	ENSG00000231304	cis	4.87x10-34	34.9	lncRNA
rs62557076	9:69449629	T	C	*RP11.146D12.2*	ENSG00000240240	trans	5.42x10-34	34.9	lncRNA
rs56402156	7:143103481	G	A	*OR2R1P*	ENSG00000236853	cis	7.86x10-34	34.8	TCunitary PG
rs7142957	14:21724070	G	A	*AP002387.1*	ENSG00000172900	trans	6.94x10-34	34.8	unitary PG
rs1637340	7:74337634	G	A	*GTF2IRD2*	ENSG00000196275	cis	1.45x10-33	34.6	PC
rs7146961	14:106993798	C	T	*IGHV3.48*	ENSG00000211964	cis	1.89x10-33	34.5	IG V gene
rs7952890	12:9478318	C	G	*RP13.735L24.1*	ENSG00000260423	cis	1.89x10-33	34.5	lncRNA
rs2505811	10:42872833	G	C	*RP11.313J2.1*	ENSG00000215146	cis	4.63x10-33	34.2	TCunPPG
rs982085	5:137775581	C	G	*KDM3B*	ENSG00000120733	cis	8.47x10-33	34	PC
rs522042	1:74666274	C	T	*RP1.20N18.6*	ENSG00000224308	trans	2.52x10-32	33.7	lncRNA
rs72642336	4:68466995	C	A	*TMPRSS11D*	ENSG00000153802	cis	2.79x10-32	33.6	PC
rs2975065	7:38401522	A	G	*TRGV4*	ENSG00000211698	cis	3.91x10-32	33.5	TR V gene
rs56350361	11:64156776	A	T	*AP003774.6*	ENSG00000231680	cis	9.39x10-32	33.3	PC
rs6725743	2:68622990	T	C	*PLEK*	ENSG00000115956	cis	9.19x10-32	33.3	lncRNA
rs2945641	11:93807605	A	C	*HEPHL1*	ENSG00000181333	cis	9.58x10-32	33.2	PC
rs1803262	2:87068827	G	A	*AC108868.4*	ENSG00000254126	trans	1.92x10-31	33	PC
rs3803798	17:7460517	G	C	*TNFSF12.TNFSF13*	ENSG00000248871	cis	1.94x10-31	33	PC
rs10902267	11:1007831	T	C	*AP2A2*	ENSG00000183020	cis	3.43x10-31	32.8	PC
rs7960229	12:93717719	T	C	*RP11.511B23.2*	ENSG00000257322	cis	3.42x10-31	32.8	lncRNA
rs684552	1:120917635	G	A	*NBPF8P*	ENSG00000270231	cis	5.66x10-31	32.7	TCunPPG
rs1777459	1:108584999	G	C	*RP11.356N1.2*	ENSG00000226822	cis	6.36x10-31	32.6	PC
rs2523590	6:31327064	T	C	*HCG4P3*	ENSG00000237669	trans	7.37x10-31	32.6	lncRNA
rs669340	17:18263957	G	C	*SHMT1*	ENSG00000176974	cis	6.08x10-31	32.6	unPPG
rs2732617	17:44363632	A	C	*KANSL1*	ENSG00000120071	cis	1.04x10-30	32.5	PC
rs17817950	17:53045325	G	A	*STXBP4*	ENSG00000166263	cis	2.71x10-30	32.2	PC
rs9611170	22:39784845	G	C	*SYNGR1*	ENSG00000100321	cis	3.08x10-30	32.1	PC
rs10448	19:59093239	T	C	*RPL23AP79*	ENSG00000213753	cis	5.39x10-30	31.9	PC
rs12184651	13:21329029	G	A	*EEF1AKMT1*	ENSG00000150456	cis	6.06x10-30	31.9	PC
rs17552066	12:27863531	T	C	*RP11.1060J15.4*	ENSG00000256377	cis	5.84x10-30	31.9	TCPPG
rs6806332	3:125763566	A	G	*ROPN1B*	ENSG00000114547	cis	5.27x10-30	31.9	lncRNA
rs3757093	6:2766779	G	A	*MYLK4*	ENSG00000145949	cis	7.14x10-30	31.8	PC
rs12798684	11:5215042	C	T	*AC104389.16*	ENSG00000224091	cis	1.42x10-29	31.6	lncRNA
rs3918010	1:36944462	A	G	*CBLN2*	ENSG00000141668	trans	2.79x10-29	31.4	PC
rs1808209	17:46140291	C	T	*HOXB2*	ENSG00000173917	cis	5.32x10-29	31.2	PC
rs143043168	19:22857004	A	G	*ZNF492*	ENSG00000229676	cis	7.19x10-29	31.1	PG
rs75045569	7:143109208	T	G	*OR10AC1P*	ENSG00000176510	cis	5.69x10-29	31.1	PC

eQTL rs ID	eQTL chr:pos	ref	alt	Gene symbol	Ensembl gene ID	cis/trans	eQTL P-value	R^2	Gene type
rs17180100	6:29936065	C	T	*HLA.G*	ENSG00000204632	cis	1.59x10-28	30.8	PC
rs10081693	9:94966486	T	G	*OGN*	ENSG00000106809	cis	2.31x10-28	30.7	PC
rs12124948	1:150527294	C	T	*HORMAD1*	ENSG00000143452	cis	1.76x10-28	30.7	PC
rs10772397	12:11138683	C	T	*TAS2R50*	ENSG00000212126	cis	2.98x10-28	30.6	PC
rs763271	22:27043233	C	G	*CTA.211A9.5*	ENSG00000244625	cis	3.05x10-28	30.6	lncRNA
rs4785093	16:60083019	A	G	*RP11.430C1.1*	ENSG00000261807	cis	3.98x10-28	30.5	lncRNA
rs3792988	6:24876260	T	C	*RP5.1112D6.5*	ENSG00000229276	trans	5.41x10-28	30.4	lncRNA
rs73046589	12:10944122	T	C	*RP11.711K1.7*	ENSG00000255790	cis	4.79x10-28	30.4	lncRNA
rs112534607	17:44344376	G	A	*AC126544.4*	ENSG00000204650	cis	6.6x10-28	30.3	TCunitary PG
rs62282782	3:195687736	G	C	*AC069513.3*	ENSG00000242086	cis	6.97x10-28	30.3	lncRNA
rs2844827	6:29811237	T	C	*HCG4P5*	ENSG00000227766	cis	9.03x10-28	30.2	unPPG
rs67933574	12:11226237	A	G	*TAS2R64P*	ENSG00000256274	cis	7.7x10-28	30.2	TCunPPG
rs10794309	11:325948	C	T	*RP11.326C3.15*	ENSG00000270972	cis	1.06x10-27	30.1	lncRNA
rs4658519	1:243110266	C	T	*CEP170P1*	ENSG00000154608	trans	1.69x10-27	30	TCunPPG
rs1133222	10:52516665	C	T	*ASAH2*	ENSG00000188611	cis	9.34x10-27	29.4	PC
rs9894225	17:4546559	A	G	*ALOX15*	ENSG00000161905	cis	9.14x10-27	29.4	PC
rs1043314	11:118915944	A	G	*HYOU1*	ENSG00000149428	cis	1.75x10-26	29.1	PC
rs11914110	22:23660328	G	A	*AP000550.1*	ENSG00000169668	trans	1.74x10-26	29.1	TCunPPG
rs2316770	17:43934256	G	A	*LRRC37A3*	ENSG00000176809	trans	2.1x10-26	29.1	PC
rs9657894	7:150437927	C	T	*RP5.1051J4.6*	ENSG00000177590	cis	2.82x10-26	29	PPG
rs146432789	5:178453100	A	G	*ZNF879*	ENSG00000234284	cis	4.33x10-26	28.8	TCunPPG
rs6465906	7:102787381	A	C	*DPY19L2P2*	ENSG00000170629	cis	4.21x10-26	28.8	PC
rs1637340	7:74337634	G	A	*AC004166.5*	ENSG00000123965	cis	5.47x10-26	28.7	PC
rs57890152	5:149821472	T	C	*RP11.343H5.4*	ENSG00000224114	trans	6.4x10-26	28.7	unPPG
rs72642336	4:68466995	C	A	*GNRHR*	ENSG00000109163	cis	6.24x10-26	28.7	PPG
rs13081249	3:152092101	T	C	*RP11.513O17.2*	ENSG00000253301	trans	9.68x10-26	28.5	lncRNA
rs373427274	5:177218466	C	T	*FAM153C*	ENSG00000204677	cis	1.23x10-25	28.4	lncRNA
rs10907940	9:42191070	C	A	*RP11.204M4.2*	ENSG00000204837	cis	1.5x10-25	28.3	TCunPPG
rs10907940	9:42191070	C	A	*RP11.111F5.5*	ENSG00000227449	trans	1.86x10-25	28.3	TCunPPG
rs143820282	1:1676091	C	T	*RP1.283E3.8*	ENSG00000268575	cis	1.57x10-25	28.3	lncRNA
rs2008345	14:23401442	T	A	*RP11.298I3.1*	ENSG00000257285	cis	1.86x10-25	28.3	TCunPPG
rs62027001	15:102317025	T	C	*CTD.2611K5.6*	ENSG00000259660	cis	1.47x10-25	28.3	lncRNA
rs905707	17:72563644	A	G	*CTD.2006K23.1*	ENSG00000261222	cis	1.57x10-25	28.3	lncRNA
rs2685190	3:130564579	G	A	*NEK11*	ENSG00000114670	cis	3.05x10-25	28.1	PC
rs72929707	2:196439583	C	T	*AC064834.2*	ENSG00000223466	cis	4.24x10-25	28	PC
rs78821460	5:127874149	G	C	*FBN2*	ENSG00000138829	cis	3.77x10-25	28	lncRNA
rs13255886	8:71565954	G	T	*XKR9*	ENSG00000221947	cis	4.93x10-25	27.9	PC
rs2836200	21:39593581	G	A	*AP001434.2*	ENSG00000226012	cis	5.35x10-25	27.9	PC
rs6933976	6:28969522	G	A	*ZNF311*	ENSG00000197935	cis	5.62x10-25	27.9	lncRNA
rs10794309	11:325948	C	T	*RP11.326C3.12*	ENSG00000255328	cis	6.15x10-25	27.8	PC
rs988067	8:133791175	C	G	*TG*	ENSG00000042832	cis	5.79x10-25	27.8	lncRNA
rs79379134	16:30292500	A	T	*RP11.345J4.5*	ENSG00000261740	cis	9.43x10-25	27.7	PC
rs11040487	11:6086071	C	T	*CTD.2008A1.2*	ENSG00000259479	trans	2.27x10-24	27.3	PC
rs114586219	15:82537939	C	A	*FAM154B*	ENSG00000188659	cis	2.14x10-24	27.3	PC

eQTL rs ID	eQTL chr:pos	ref	alt	Gene symbol	Ensembl gene ID	cis/trans	eQTL P-value	R^2	Gene type
rs2982122	1:148002289	C	T	*NBPF26*	ENSG00000273136	trans	2.72x10-24	27.3	PC
rs324122	19:52893957	C	T	*ZNF880*	ENSG00000221923	cis	2.43x10-24	27.3	TCunPPG
rs3366	21:44834825	G	A	*SIK1*	ENSG00000142178	cis	2.2x10-24	27.3	PC
rs1985275	11:9000993	A	G	*TMEM9B.AS1*	ENSG00000254860	cis	3.41x10-24	27.2	PC
rs75364523	17:4723121	G	A	*CHRNE*	ENSG00000108556	cis	3.22x10-24	27.2	lncRNA
rs1056739	10:104236542	T	C	*MFSD13A*	ENSG00000138111	cis	3.87x10-24	27.1	TCunPPG
rs4267298	16:70004028	C	T	*CLEC18B*	ENSG00000140839	trans	4.39x10-24	27.1	PC
rs7791024	7:66766024	T	C	*PMS2L4*	ENSG00000067601	cis	3.75x10-24	27.1	PC
rs11099650	4:85483950	A	C	*AC005022.1*	ENSG00000224017	trans	5.76x10-24	27	PC
rs12269882	11:79092486	A	G	*ODZ4*	ENSG00000149256	cis	4.87x10-24	27	lncRNA
rs7952790	12:68406452	C	T	*GS1.410F4.3*	ENSG00000236946	cis	5.03x10-24	27	TCPPG
rs1892568	21:39563599	T	C	*AP001434.1*	ENSG00000231123	cis	6.26x10-24	26.9	PC
rs4050880	22:22624189	C	A	*IGLV5.52*	ENSG00000211643	cis	6.56x10-24	26.9	lncRNA
rs56006248	17:43815141	A	G	*LRRC37A*	ENSG00000176681	cis	7.47x10-24	26.9	PC
rs639455	1:20978221	C	T	*RP11.401M16.7*	ENSG00000117242	cis	7.37x10-24	26.9	IG V gene
rs7872739	9:95217930	C	T	*ECM2*	ENSG00000106823	cis	6.58x10-24	26.9	PPG
rs3894776	19:1457370	C	T	*CTB.25B13.12*	ENSG00000267317	cis	1.1x10-23	26.7	lncRNA
rs10221200	17:15589734	G	A	*TRIM16*	ENSG00000221926	cis	1.67x10-23	26.6	PC
rs1501371	15:82460440	A	C	*UBE2Q2P2*	ENSG00000259429	cis	1.49x10-23	26.6	TCPPG
rs375407631	9:66470329	A	G	*RP11.262H14.7*	ENSG00000227582	cis	1.5x10-23	26.6	TCunPPG
rs4006668	2:219595001	C	T	*RP11.459I19.1*	ENSG00000272555	cis	1.87x10-23	26.5	PC
rs77727254	17:30986984	C	T	*TMEM98*	ENSG00000006042	cis	2.08x10-23	26.5	PC
rs9926	4:10076860	G	A	*WDR1*	ENSG00000071127	cis	2.02x10-23	26.5	lncRNA
rs10470179	21:39570684	C	T	*AC079753.4*	ENSG00000180152	trans	2.27x10-23	26.4	PPG
rs13340529	7:66466119	A	C	*TYW1*	ENSG00000198874	cis	2.31x10-23	26.4	PC
rs2641419	11:4555193	A	C	*OR52I2*	ENSG00000226288	cis	2.25x10-23	26.4	lncRNA
rs7898547	10:6659210	A	C	*RP11.554I8.1*	ENSG00000225948	cis	2.58x10-23	26.4	PC
rs3917945	1:36942233	T	G	*CA5A*	ENSG00000174990	trans	3.21x10-23	26.3	PC
rs9264682	6:31240872	T	G	*HLA.K*	ENSG00000230795	trans	3.48x10-23	26.3	unPPG
rs140950438	5:59983635	G	C	*DEPDC1B*	ENSG00000035499	cis	4.59x10-23	26.2	PC
rs35117338	7:66072781	A	T	*GS1.124K5.12*	ENSG00000229180	cis	4.15x10-23	26.2	PC
rs3930740	17:8294959	C	T	*KRBA2*	ENSG00000184619	cis	4.42x10-23	26.2	TCunPPG
rs4685744	3:4403537	C	T	*AC023480.1*	ENSG00000229241	cis	4.24x10-23	26.2	PPG
rs10179864	2:238675892	A	G	*RP11.255C15.1*	ENSG00000240429	trans	5.65x10-23	26.1	PC
rs112161250	15:58440089	G	GCACA	*RP1*	ENSG00000104237	trans	5.3x10-23	26.1	PC
rs2596496	6:31322782	C	G	*HLA.B*	ENSG00000234745	cis	6.12x10-23	26.1	PPG
rs113590651	8:82707315	A	G	*SNX16*	ENSG00000104497	cis	6.42x10-23	26	PC
rs1749596	1:236380913	T	C	*ERO1B*	ENSG00000086619	cis	7.55x10-23	26	PC
rs2596503	6:31320810	A	G	*DDX39B*	ENSG00000198563	cis	6.22x10-23	26	PC
rs12366	2:75185856	G	T	*AC104135.2*	ENSG00000236209	cis	1.02x10-22	25.9	lncRNA
rs3026243	12:9183400	T	C	*RP11.118B22.1*	ENSG00000256817	cis	8.09x10-23	25.9	PPG
rs2285170	1:172425529	C	G	*SUCO*	ENSG00000094975	cis	1.26x10-22	25.8	PC
rs7400769	14:94613418	A	T	*PPP4R4*	ENSG00000119698	cis	1.09x10-22	25.8	PC
rs77972028	1:149674716	G	A	*CH17.118O6.2*	ENSG00000226067	cis	1.15x10-22	25.8	lncRNA
rs7295439	12:89825122	G	T	*RP11.734K2.4*	ENSG00000270344	cis	1.4x10-22	25.7	lncRNA

eQTL rs ID	eQTL chr:pos	ref	alt	Gene symbol	Ensembl gene ID	cis/trans	eQTL P-value	R^2	Gene type
rs2435201	17:44078982	T	A	*RP11.995C19.3*	ENSG00000232300	cis	1.89x10-22	25.6	PC
rs6502892	17:1617608	T	C	*TLCD2*	ENSG00000185561	cis	2.17x10-22	25.6	PC
rs7096229	10:91394900	T	G	*RP11.248C1.2*	ENSG00000232229	cis	1.75x10-22	25.6	lncRNA
rs72697606	8:145611531	A	T	*CPSF1*	ENSG00000071894	cis	1.75x10-22	25.6	lncRNA
rs11657128	17:56048840	G	A	*RP11.415I12.3*	ENSG00000249753	trans	2.59x10-22	25.5	lncRNA
rs4013507	10:48303741	G	A	*RP11.144G6.12*	ENSG00000254929	trans	3.35x10-22	25.4	lncRNA
rs76549974	9:43793814	C	T	*RP11.146D12.2*	ENSG00000240240	trans	3.21x10-22	25.4	lncRNA
rs17442763	12:40538796	T	A	*RP11.476D10.1*	ENSG00000260943	cis	4.33x10-22	25.3	PC
rs3117640	19:55338833	G	A	*KIR3DL1*	ENSG00000167633	cis	4.03x10-22	25.3	PC
rs368785114	9:45729667	C	A	*RP11.146D12.2*	ENSG00000240240	trans	3.94x10-22	25.3	lncRNA
rs7838905	8:2954809	C	G	*CSMD1*	ENSG00000183117	cis	4.47x10-22	25.3	lncRNA
rs2523815	6:29840243	A	G	*HLA.K*	ENSG00000230795	cis	5.44x10-22	25.2	PC
rs3087563	11:117707937	T	C	*FXYD6*	ENSG00000137726	cis	5.38x10-22	25.2	lncRNA
rs6488334	12:11140444	T	C	*TAS2R46*	ENSG00000226761	cis	5.15x10-22	25.2	PC
rs8073963	17:46609918	G	A	*HOXB.AS1*	ENSG00000230148	cis	5.01x10-22	25.2	lncRNA
rs9368476	6:26906789	A	G	*RP11.457M11.5*	ENSG00000261584	cis	5.45x10-22	25.2	unPPG
rs995059	9:114439510	C	T	*RP11.18M11.2*	ENSG00000224812	trans	5.88x10-22	25.2	lncRNA
rs1852270	17:43655280	G	T	*LRRC37A*	ENSG00000176681	cis	7.65x10-22	25.1	PC
rs2523815	6:29840243	A	G	*HCG4B*	ENSG00000227262	cis	6.32x10-22	25.1	PC
rs62050500	17:21446432	G	A	*C17orf51*	ENSG00000212719	cis	6.93x10-22	25.1	lncRNA
rs6720375	2:132440073	G	C	*AP000525.9*	ENSG00000206195	trans	7.08x10-22	25.1	lncRNA
rs7480474	11:307912	T	C	*IFITM3*	ENSG00000142089	cis	6.29x10-22	25.1	unPPG
rs12489198	3:193294307	G	T	*RP11.175P19.2*	ENSG00000236297	cis	9.45x10-22	25	PC
rs8068200	17:45390562	A	G	*RP11.66B24.7*	ENSG00000272808	trans	9.41x10-22	25	TCPPG
rs8077275	17:18705412	G	A	*TVP23B*	ENSG00000171928	cis	8.76x10-22	25	lncRNA
rs12763558	10:70872970	G	A	*VPS26A*	ENSG00000122958	cis	1.36x10-21	24.8	PC
rs879985390	7:101977597	G	A	*LRWD1*	ENSG00000161036	cis	1.36x10-21	24.8	PC
rs13401870	2:68340446	A	G	*CNRIP1*	ENSG00000119865	cis	1.82x10-21	24.7	PC
rs1678185	2:231409854	C	T	*UNC13C*	ENSG00000137766	trans	2.25x10-21	24.6	PC
rs1834274	2:131951961	G	A	*NF1L2*	ENSG00000236956	cis	2.38x10-21	24.6	unPPG
rs2388419	15:94400712	G	A	*RP11.76E17.4*	ENSG00000258831	cis	2.38x10-21	24.6	lncRNA
rs10995796	10:51592744	T	G	*PARGP1*	ENSG00000239883	trans	3.23x10-21	24.5	TCunPPG
rs58304714	1:150831561	T	A	*RP11.732A21.2*	ENSG00000260008	trans	3.31x10-21	24.5	lncRNA
rs72836318	17:44121579	T	C	*RP11.798G7.8*	ENSG00000266918	cis	2.91x10-21	24.5	lncRNA
rs7897787	10:18921314	C	A	*RP11.499P20.2*	ENSG00000240291	cis	2.97x10-21	24.5	TEC
rs11648223	16:29585333	C	A	*RP11.231C14.3*	ENSG00000254634	cis	3.56x10-21	24.4	unPPG
rs12475433	2:173293419	G	T	*AC078883.4*	ENSG00000226963	cis	5.69x10-21	24.2	PC
rs2490849	9:15388507	C	T	*CCDC171*	ENSG00000164989	cis	5.6x10-21	24.2	PC
rs2753747	10:81512832	A	G	*NUTM2B*	ENSG00000188199	cis	5.76x10-21	24.2	PC
rs2908776	16:53407506	C	G	*RP11.44F14.2*	ENSG00000261804	cis	5.58x10-21	24.2	PC
rs72744331	9:76788027	T	C	*RORB*	ENSG00000198963	cis	6.04x10-21	24.2	lncRNA
rs7947900	11:316299	T	G	*IFITM2*	ENSG00000185201	cis	6.28x10-21	24.2	lncRNA
rs430748	22:18896529	C	T	*DGCR6*	ENSG00000183628	cis	8.04x10-21	24.1	PC
rs9761856	4:119579892	G	A	*SYNPO2*	ENSG00000172403	cis	7.48x10-21	24.1	PC
rs12653907	5:175485237	C	A	*RP11.844P9.5*	ENSG00000248596	cis	1.07x10-20	24	PC

eQTL rs ID	eQTL chr:pos	ref	alt	Gene symbol	Ensembl gene ID	cis/trans	eQTL P-value	R^2	Gene type
rs190206	17:15464679	G	A	*TVP23C.CDRT4*	ENSG00000259024	cis	1.03x10-20	24	PC
rs6925979	6:142651052	G	A	*ADGRG6*	ENSG00000112414	cis	9.91x10-21	24	lncRNA
rs876554	12:89885473	T	C	*POC1B*	ENSG00000139323	cis	9.87x10-21	24	PC
rs2377220	1:1660635	T	G	*CDK11A*	ENSG00000008128	cis	1.3x10-20	23.9	PC
rs3118000	1:16909820	C	G	*MST1L*	ENSG00000186715	cis	1.28x10-20	23.9	PC
rs7273242	20:62594160	T	C	*SAMD10*	ENSG00000130590	cis	1.41x10-20	23.9	TCunPPG
rs72836318	17:44121579	T	C	*ARL17B*	ENSG00000228696	cis	1.18x10-20	23.9	PC
rs11619622	13:42570201	A	G	*VWA8*	ENSG00000102763	cis	1.81x10-20	23.8	PC
rs3095174	17:16254494	C	G	*CENPV*	ENSG00000166582	cis	1.62x10-20	23.8	PC
rs3098394	19:12397460	G	A	*CTD.2666L21.2*	ENSG00000234750	cis	1.45x10-20	23.8	PC
rs4236478	7:142411527	T	C	*U66061.35*	ENSG00000244273	cis	1.56x10-20	23.8	PPG
rs4484184	3:53981048	T	C	*ACTR8*	ENSG00000113812	cis	1.62x10-20	23.8	PPG
rs12975429	19:33199227	G	A	*NUDT19*	ENSG00000213965	cis	1.97x10-20	23.7	PC
rs3102342	16:89451575	T	C	*RP1.168P16.1*	ENSG00000261692	cis	1.96x10-20	23.7	lncRNA
rs6960171	7:64910100	G	A	*RP11.792A8.4*	ENSG00000272831	cis	1.95x10-20	23.7	lncRNA
rs1042229	19:52249672	A	C	*FPR1*	ENSG00000171051	cis	2.34x10-20	23.6	PC
rs7829886	8:95497388	T	A	*KIAA1429*	ENSG00000164944	cis	2.53x10-20	23.6	PC
rs2373340	7:148001246	A	G	*CNTNAP2*	ENSG00000174469	trans	3.68x10-20	23.5	PC
rs1375862	9:125617037	G	A	*ZBTB26*	ENSG00000171448	cis	4.57x10-20	23.4	PC
rs72689277	4:185593699	G	A	*PRIMPOL*	ENSG00000164306	cis	4.62x10-20	23.4	PC
rs1051664	1:45116985	C	T	*RNF220*	ENSG00000187147	cis	5.82x10-20	23.3	PC
rs2003490	14:75377555	C	A	*DLST*	ENSG00000119689	cis	5.97x10-20	23.3	PC
rs7350611	12:11331094	T	C	*TAS2R13*	ENSG00000212128	cis	5.99x10-20	23.3	PC
rs112413396	4:70278401	T	C	*UGT2B28*	ENSG00000135226	cis	7.37x10-20	23.2	PC
rs28652789	16:83807	G	C	*DDX11L10*	ENSG00000233614	cis	6.36x10-20	23.2	PC
rs56104184	19:49370310	C	T	*PPP1R15A*	ENSG00000087074	cis	6.84x10-20	23.2	TCunPPG
rs12704823	7:76822127	C	T	*AC073635.5*	ENSG00000250990	cis	8.66x10-20	23.1	PC
rs867663893	3:122443492	A	T	*DIRC2*	ENSG00000138463	cis	8.2x10-20	23.1	lncRNA
rs13333072	16:8823848	A	G	*ABAT*	ENSG00000183044	cis	1.12x10-19	23	PC
rs142929928	17:4783340	C	G	*C17orf107*	ENSG00000205710	cis	1.08x10-19	23	PPG
rs2286466	16:2014283	A	G	*RPS2P46*	ENSG00000189343	trans	1.21x10-19	23	PC
rs6085271	20:5748056	T	C	*C20orf196*	ENSG00000171984	cis	1.29x10-19	22.9	PC
rs987534	8:120713652	G	A	*DSCC1*	ENSG00000136982	cis	1.48x10-19	22.9	PC
rs9796412	14:77211737	C	T	*RP11.488C13.5*	ENSG00000258301	cis	1.64x10-19	22.8	lncRNA
rs11076704	16:88777995	C	T	*CTU2*	ENSG00000174177	cis	2.33x10-19	22.7	PC
rs1355775	6:52529358	G	A	*RP1.152L7.5*	ENSG00000216775	cis	2.05x10-19	22.7	PC
rs76312359	15:55829689	A	T	*PYGO1*	ENSG00000171016	cis	2.14x10-19	22.7	PC
rs9514837	13:108995148	G	C	*MYO16*	ENSG00000041515	cis	1.99x10-19	22.7	TCunPPG
rs12167058	22:29907401	C	T	*RP11.638L3.3*	ENSG00000265555	trans	2.65x10-19	22.6	PC
rs2769345	6:167366394	T	C	*RP11.514O12.4*	ENSG00000249141	cis	2.7x10-19	22.6	PC
rs35940311	1:93663604	G	A	*DR1*	ENSG00000117505	cis	2.7x10-19	22.6	PC
rs9272779	6:32610314	T	C	*HLA.DQA1*	ENSG00000196735	cis	2.64x10-19	22.6	lncRNA
rs162298	6:167455629	G	A	*NPTXR*	ENSG00000221890	trans	3.28x10-19	22.5	PC
rs58581511	17:57950621	T	G	*RNFT1*	ENSG00000189050	cis	3.39x10-19	22.5	PC
rs2588347	12:10944783	A	G	*TAS2R31*	ENSG00000256436	cis	4.88x10-19	22.4	PC

eQTL rs ID	eQTL chr:pos	ref	alt	Gene symbol	Ensembl gene ID	cis/trans	eQTL P-value	R^2	Gene type
rs7718927	5:109907035	A	T	*TMEM232*	ENSG00000186952	cis	3.99x10-19	22.4	PC
rs12216750	8:82066711	T	A	*RP11.1149M10.2*	ENSG00000253214	cis	7.12x10-19	22.2	PC
rs12464694	2:196908927	A	G	*DNAH7*	ENSG00000118997	cis	6.41x10-19	22.2	PC
rs2924251	17:46338677	A	G	*HOXB3*	ENSG00000120093	cis	6.45x10-19	22.2	PC
rs4674336	2:219632820	A	G	*PRKAG3*	ENSG00000115592	cis	6.31x10-19	22.2	lncRNA
rs586746	19:53080928	A	G	*ZNF701*	ENSG00000167562	cis	9.73x10-19	22.1	PC
rs1873379	15:75498744	G	C	*RP11.817O13.8*	ENSG00000260274	cis	1.22x10-18	22	PC
rs2302058	19:57703077	T	G	*ZNF264*	ENSG00000083844	cis	1.15x10-18	22	PPG
rs2832328	21:30821767	C	G	*RP11.313P22.1*	ENSG00000273464	cis	9.97x10-19	22	lncRNA
rs6589587	11:116875338	T	G	*AP000936.1*	ENSG00000234268	cis	1.16x10-18	22	lncRNA
rs10400906	15:83297138	C	T	*CPEB1*	ENSG00000214575	cis	1.43x10-18	21.9	PC
rs2780681	10:27512767	A	G	*RP11.85G18.4*	ENSG00000230445	cis	1.29x10-18	21.9	PC
rs61148413	15:84828326	T	A	*GOLGA6L4*	ENSG00000184206	cis	1.48x10-18	21.9	PC
rs7223043	17:33826654	A	G	*RP11.1094M14.8*	ENSG00000267369	cis	1.37x10-18	21.9	TCPPG
rs8058597	16:70082214	G	C	*NPIPB15*	ENSG00000196436	trans	1.44x10-18	21.9	PPG
rs145475856	1:146367867	G	T	*PFN1P8*	ENSG00000244371	cis	1.66x10-18	21.8	PC
rs62371990	5:42604782	G	A	*CCDC152*	ENSG00000198865	cis	1.76x10-18	21.8	PPG
rs2588347	12:10944783	A	G	*TAS2R12*	ENSG00000256682	cis	2.05x10-18	21.7	lncRNA
rs964611	15:48597514	C	A	*RP5.898J17.1*	ENSG00000231987	trans	2.43x10-18	21.7	TCPPG
rs7223732	17:45881197	G	C	*SCRN2*	ENSG00000141295	cis	3.08x10-18	21.6	PC
rs9893390	17:33751315	T	A	*RP11.686D22.3*	ENSG00000267648	cis	3.09x10-18	21.6	TCPPG
rs10079553	5:178959047	C	T	*RUFY1*	ENSG00000176783	cis	3.54x10-18	21.5	PC
rs12728538	1:63959125	A	C	*ITGB3BP*	ENSG00000142856	cis	3.25x10-18	21.5	PC
rs12939821	17:62079286	G	A	*PRR29*	ENSG00000224383	cis	3.75x10-18	21.5	PC
rs200276266	5:175530214	C	T	*FAM153A*	ENSG00000170074	trans	3.13x10-18	21.5	PC
rs4744150	9:95630363	C	T	*RP11.526D8.3*	ENSG00000226721	cis	3.44x10-18	21.5	PC
rs78042407	10:49333147	G	C	*AGAP9*	ENSG00000204172	trans	3.35x10-18	21.5	PPG
rs10125553	9:37841380	A	G	*DCAF10*	ENSG00000122741	cis	4.48x10-18	21.4	PC
rs144921768	16:32973407	G	A	*DUSP22*	ENSG00000112679	trans	5.91x10-18	21.3	PC
rs2450556	8:95330332	A	C	*FSBP*	ENSG00000265817	cis	7.31x10-18	21.2	PC
rs4083651	9:19211852	A	G	*RP11.513M16.8*	ENSG00000273226	cis	7.64x10-18	21.2	lncRNA
rs11659948	18:9197372	A	G	*RP11.63E9.1*	ENSG00000259828	trans	9.25x10-18	21.1	PC
rs12718958	7:55431162	A	G	*LANCL2*	ENSG00000132434	cis	8.91x10-18	21.1	lncRNA
rs2873872	17:49508445	A	T	*RP11.1018N14.5*	ENSG00000267452	cis	8.46x10-18	21.1	lncRNA
rs6558530	8:1706207	A	G	*AC093375.1*	ENSG00000226383	trans	8.42x10-18	21.1	lncRNA
rs116918202	16:89587080	C	T	*CDK10*	ENSG00000185324	cis	1.17x10-17	21	PC
rs1817303	10:51450060	A	C	*RP11.546J1.1*	ENSG00000270557	trans	1.05x10-17	21	lncRNA
rs9412607	9:42901002	T	G	*RP11.745L13.2*	ENSG00000251555	trans	1.16x10-17	21	lncRNA
rs10405910	19:57961191	A	T	*AC004076.9*	ENSG00000268163	cis	1.43x10-17	20.9	PC
rs179468	22:24109774	T	G	*CHCHD10*	ENSG00000250479	cis	1.49x10-17	20.9	lncRNA
rs2671827	17:33732330	C	T	*RP11.686D22.8*	ENSG00000267745	cis	1.35x10-17	20.9	PC
rs73153794	7:134853085	A	C	*RP11.90K6.1*	ENSG00000239482	trans	1.47x10-17	20.9	lncRNA
rs9353446	6:87780724	T	C	*C6orf163*	ENSG00000203872	cis	1.36x10-17	20.9	PC
rs111277130	5:1634070	C	T	*CTD.2083E4.4*	ENSG00000260774	trans	1.62x10-17	20.8	PC
rs1128894	1:153604422	T	C	*S100A1*	ENSG00000160678	cis	1.66x10-17	20.8	PC

eQTL rs ID	eQTL chr:pos	ref	alt	Gene symbol	Ensembl gene ID	cis/trans	eQTL P-value	R^2	Gene type
rs1367224	2:175391901	T	C	*AC018890.6*	ENSG00000236449	cis	1.54x10-17	20.8	PC
rs67425176	8:66543064	T	C	*CTD.3025N20.3*	ENSG00000272010	cis	1.76x10-17	20.8	lncRNA
rs6816018	4:88735959	G	C	*SPP1*	ENSG00000118785	cis	1.76x10-17	20.8	lncRNA
rs73213579	4:198953	C	A	*SOHLH2*	ENSG00000120669	trans	1.55x10-17	20.8	lncRNA
rs1007591	11:35731800	T	G	*TRIM44*	ENSG00000166326	cis	2.05x10-17	20.7	PC
rs11009104	10:33116039	A	G	*CCDC7*	ENSG00000216937	cis	1.89x10-17	20.7	PC
rs13081249	3:152092101	T	C	*ANO7*	ENSG00000146205	trans	2.22x10-17	20.7	PC
rs529150802	16:89268323	G	T	*FANCA*	ENSG00000187741	cis	2.14x10-17	20.7	PC
rs11150958	18:74248237	A	G	*RP11.17M16.2*	ENSG00000265778	cis	2.56x10-17	20.6	lncRNA
rs2075458	7:130528147	A	G	*AC058791.1*	ENSG00000226380	cis	2.68x10-17	20.6	lncRNA
rs2732617	17:44363632	A	C	*MKRN9P*	ENSG00000258128	trans	2.57x10-17	20.6	TCPPG
rs591411	9:79313529	C	T	*PCA3*	ENSG00000225937	cis	2.84x10-17	20.6	lncRNA
rs62469316	7:64674302	T	G	*RP11.792A8.4*	ENSG00000272831	trans	2.47x10-17	20.6	lncRNA
rs2416548	12:11324176	C	A	*SMIM10L1*	ENSG00000256537	cis	3.19x10-17	20.5	PC
rs4472162	4:79626160	A	T	*RP11.109G23.3*	ENSG00000260278	cis	3.41x10-17	20.5	lncRNA
rs1541533	11:17096211	A	G	*SNORD14A*	ENSG00000272034	cis	4.41x10-17	20.4	PC
rs1967415	17:57113124	C	A	*TRIM37*	ENSG00000108395	cis	4.04x10-17	20.4	PC
rs1982074	19:10668673	A	G	*KRI1*	ENSG00000129347	cis	4.16x10-17	20.4	PC
rs373769561	9:41922713	G	C	*RP11.262H14.3*	ENSG00000234665	trans	3.89x10-17	20.4	lncRNA
rs7105835	11:78075725	T	C	*RP11.452H21.1*	ENSG00000254420	cis	3.74x10-17	20.4	lncRNA
rs7837964	8:22264417	G	C	*SLC39A14*	ENSG00000104635	cis	3.8x10-17	20.4	snoRNA
rs11078300	17:2571489	G	A	*PAFAH1B1*	ENSG00000007168	cis	5.31x10-17	20.3	PC
rs11249686	5:177181014	A	G	*FAM153B*	ENSG00000182230	trans	5.13x10-17	20.3	PC
rs2730272	7:158798622	G	C	*LINC00689*	ENSG00000231419	cis	4.96x10-17	20.3	PC
rs492095	17:72869078	A	G	*FDXR*	ENSG00000161513	cis	4.85x10-17	20.3	lncRNA
rs11186721	10:93641908	C	A	*AC005307.1*	ENSG00000260725	trans	6.82x10-17	20.2	PC
rs376869011	9:66530411	A	G	*FGF7*	ENSG00000140285	trans	5.72x10-17	20.2	lncRNA
rs9893390	17:33751315	T	A	*RP11.686D22.4*	ENSG00000267547	cis	6.18x10-17	20.2	lncRNA
rs12514888	5:34221989	G	A	*RP11.1023L17.2*	ENSG00000215156	cis	7.93x10-17	20.1	PPG
rs3127726	10:46932364	T	C	*RP11.144G6.12*	ENSG00000254929	cis	7.76x10-17	20.1	TCunitary PG
rs35858034	19:41299864	A	C	*CYP2T2P*	ENSG00000233622	cis	7.39x10-17	20.1	lncRNA
rs11209243	1:68738861	G	C	*RP11.518D3.3*	ENSG00000234383	cis	9.9x10-17	20	PC
rs11799352	1:118576627	A	T	*SPAG17*	ENSG00000155761	cis	1.06x10-16	20	TCPPG
rs150134394	10:81455053	T	C	*RP11.119F19.4*	ENSG00000224886	cis	1.09x10-16	20	PPG
rs4968290	17:45047384	C	T	*RP11.156P1.3*	ENSG00000262879	cis	1.02x10-16	20	unPPG
rs61920361	12:27165921	A	T	*RP11.582E3.4*	ENSG00000256625	cis	9x10-17	20	lncRNA
rs10752555	9:70484945	A	G	*RP11.262H14.3*	ENSG00000234665	trans	1.23x10-16	19.9	PC
rs4624506	3:39444901	T	C	*SLC25A38*	ENSG00000144659	cis	1.29x10-16	19.9	PC
rs55962832	3:40244451	T	C	*ENTPD3*	ENSG00000168032	cis	1.34x10-16	19.9	lncRNA
rs10772397	12:11138683	C	T	*TAS2R19*	ENSG00000212124	cis	1.43x10-16	19.8	PC
rs34511016	5:79881909	A	G	*FAM151B*	ENSG00000152380	cis	1.59x10-16	19.8	PC
rs677078	11:121432287	C	A	*RP11.86H7.1*	ENSG00000224081	trans	1.58x10-16	19.8	PC
rs72642346	4:68479934	C	T	*AC079880.1*	ENSG00000232398	cis	1.6x10-16	19.8	PC
rs7839535	8:134586637	A	G	*RP11.629O1.2*	ENSG00000261220	cis	1.54x10-16	19.8	TCPPG

eQTL rs ID	eQTL chr:pos	ref	alt	Gene symbol	Ensembl gene ID	cis/trans	eQTL P-value	R^2	Gene type
rs9353446	6:87780724	T	C	*SMIM8*	ENSG00000111850	cis	1.61x10-16	19.8	unPPG
NA	17:80735913	C	CCTCA	*ARL16*	ENSG00000214087	trans	1.69x10-16	19.8	lncRNA
rs132650	22:36554588	C	T	*CADPS*	ENSG00000163618	trans	1.72x10-16	19.7	PC
rs2388419	15:94400712	G	A	*RP11.76E17.3*	ENSG00000258476	cis	2.05x10-16	19.7	PPG
rs4585080	2:99557327	T	C	*AC019097.7*	ENSG00000231822	cis	1.74x10-16	19.7	lncRNA
rs71409258	15:80159117	A	G	*ST20.MTHFS*	ENSG00000259332	cis	1.85x10-16	19.7	PC
rs10771996	12:9811352	A	G	*AC003005.2*	ENSG00000268266	trans	2.16x10-16	19.6	PC
rs2008758	17:19893590	C	T	*AKAP10*	ENSG00000108599	cis	2.15x10-16	19.6	PC
rs2531805	6:28412326	C	T	*ZSCAN23*	ENSG00000187987	cis	2.16x10-16	19.6	PC
rs28549275	7:138886066	C	T	*LUC7L2*	ENSG00000146963	cis	2.25x10-16	19.6	PC
rs3785884	17:44057595	G	A	*LRRC37A2*	ENSG00000238083	cis	2.33x10-16	19.6	lncRNA
rs7962354	12:62823325	T	C	*RP11.769N19.2*	ENSG00000257880	cis	2.17x10-16	19.6	lncRNA
rs2309426	9:71830308	T	G	*TJP2*	ENSG00000119139	cis	3.17x10-16	19.5	PC
rs2945247	8:8096004	G	A	*ABC11.48400900C8.1*	ENSG00000254527	trans	2.78x10-16	19.5	PC
rs374559823	2:87366345	C	T	*RGPD1*	ENSG00000187627	cis	3.17x10-16	19.5	PC
rs61811182	1:183585327	A	G	*SCGB3A2*	ENSG00000164265	trans	2.9x10-16	19.5	PC
rs74651789	4:165898682	C	T	*TRIM61*	ENSG00000183439	cis	2.78x10-16	19.5	PPG
rs116044941	17:73874684	G	C	*RP11.552F3.9*	ENSG00000267801	cis	3.44x10-16	19.4	PC
rs3106150	17:62919344	G	A	*DND1P1*	ENSG00000264070	trans	3.86x10-16	19.4	PC
rs743810	22:35742925	T	G	*TOM1*	ENSG00000100284	cis	3.68x10-16	19.4	PPG
rs9895898	17:18644142	T	C	*TRIM16L*	ENSG00000108448	cis	3.64x10-16	19.4	lncRNA
rs11052771	12:9891371	C	T	*RP11.75L1.2*	ENSG00000213443	cis	4.3x10-16	19.3	PC
rs2169764	19:14193735	G	A	*CTB.55O6.8*	ENSG00000141854	cis	4.49x10-16	19.3	PPG
rs2540431	2:202367621	G	T	*AC007256.5*	ENSG00000213090	cis	4.58x10-16	19.3	PPG
rs2949585	1:148002266	A	C	*NBPF8P*	ENSG00000270231	trans	4.35x10-16	19.3	lncRNA
rs9289971	3:156389947	T	C	*RP11.791I24.4*	ENSG00000243926	cis	4.07x10-16	19.3	TCunPPG
rs7978962	12:10524824	T	C	*RP11.277P12.6*	ENSG00000255641	cis	5.07x10-16	19.2	PC
rs111527470	2:239149377	G	C	*AC016757.3*	ENSG00000186235	cis	6.53x10-16	19.1	TCunPPG
rs11912319	22:50937900	A	G	*CTA.384D8.34*	ENSG00000273272	cis	6.52x10-16	19.1	PC
rs148853830	7:99912929	A	T	*PMS2L1*	ENSG00000078319	cis	6.27x10-16	19.1	lncRNA
rs2569102	5:149800881	G	C	*RPS14*	ENSG00000164587	cis	6.29x10-16	19.1	lncRNA
rs2611491	10:51489393	T	G	*CTGLF10P*	ENSG00000230869	trans	7.32x10-16	19.1	TCPPG
rs28625723	7:135715783	T	C	*AC024084.1*	ENSG00000230649	cis	6.27x10-16	19.1	lncRNA
rs7216865	17:6891851	A	T	*AC027763.2*	ENSG00000215067	cis	7.26x10-16	19.1	unPPG
rs9834	5:176733616	T	C	*RP11.753G20.1*	ENSG00000217325	trans	7.16x10-16	19.1	lncRNA
rs509421	1:24035696	G	A	*CNR2*	ENSG00000188822	cis	8.01x10-16	19	PC
rs11048496	12:9172421	G	A	*AP000688.11*	ENSG00000236677	trans	1.15x10-15	18.9	PC
rs11640445	16:60195615	T	A	*RP11.430C1.1*	ENSG00000261807	cis	1.07x10-15	18.9	lncRNA
rs218663	17:6567190	A	G	*CTC.281F24.1*	ENSG00000261996	cis	9.55x10-16	18.9	lncRNA
rs2530839	17:28964727	C	T	*RP11.271K11.2*	ENSG00000264943	cis	1.12x10-15	18.9	lncRNA
rs4657614	1:166899261	A	G	*ILDR2*	ENSG00000143195	cis	9.94x10-16	18.9	PPG
rs12441089	15:93277514	T	C	*RP11.386M24.4*	ENSG00000258741	cis	1.32x10-15	18.8	PC
rs12482295	21:15263797	C	T	*RP11.757O6.2*	ENSG00000265787	trans	1.42x10-15	18.8	TCPPG
rs12917526	15:81025457	G	C	*FAM108C1*	ENSG00000136379	cis	1.45x10-15	18.8	TCunPPG
rs115654156	6:31395040	G	C	*MICA*	ENSG00000204520	cis	1.55x10-15	18.7	PC

eQTL rs ID	eQTL chr:pos	ref	alt	Gene symbol	Ensembl gene ID	cis/trans	eQTL P-value	R^2	Gene type
rs4649586	1:144611851	G	A	*CH17.118O6.2*	ENSG00000226067	trans	1.47x10-15	18.7	lncRNA
rs60545295	15:48521101	G	A	*CTB.43E15.3*	ENSG00000253686	trans	1.59x10-15	18.7	lncRNA
rs10136164	14:37483309	T	C	*PAX9*	ENSG00000198807	cis	1.89x10-15	18.6	PC
rs10752555	9:70484945	A	G	*FGF7*	ENSG00000140285	trans	1.9x10-15	18.6	PC
rs11655481	17:30622552	C	T	*CYP4F11*	ENSG00000171903	trans	1.85x10-15	18.6	PC
rs1808199	6:29924542	G	T	*SPINK1*	ENSG00000164266	trans	1.9x10-15	18.6	PC
rs2005594	22:19021819	A	G	*AC000095.8*	ENSG00000241527	cis	2.12x10-15	18.6	PC
rs489552	9:27196617	C	T	*RP11.56F10.3*	ENSG00000254396	cis	2.13x10-15	18.6	unitary PG
rs79710144	10:17723145	T	A	*FAT3*	ENSG00000165323	trans	1.94x10-15	18.6	lncRNA
rs2353408	3:142791030	A	C	*U2SURP*	ENSG00000163714	cis	2.48x10-15	18.5	PC
rs34499924	11:60970328	A	G	*PGA3*	ENSG00000229859	cis	2.58x10-15	18.5	lncRNA
rs375809946	5:175499074	G	A	*FAM153C*	ENSG00000204677	trans	2.57x10-15	18.5	PC
rs7531159	1:74653039	C	T	*FPGT.TNNI3K*	ENSG00000259030	cis	2.33x10-15	18.5	lncRNA
rs7835354	8:19564149	A	G	*RP11.1105O14.1*	ENSG00000253270	cis	2.35x10-15	18.5	PC
rs3904094	13:37683479	T	C	*VMAC*	ENSG00000187650	trans	2.86x10-15	18.4	PC
rs9949547	18:37381312	T	C	*RP11.244M2.1*	ENSG00000267374	cis	3.34x10-15	18.4	lncRNA
rs201958810	10:47097863	G	GTCTC	*AGAP9*	ENSG00000204172	cis	3.92x10-15	18.3	PC
rs3865258	17:18709492	T	C	*TVP23C*	ENSG00000175106	trans	4x10-15	18.3	PC
rs3915878	4:144915780	C	T	*RP11.248J18.2*	ENSG00000269906	trans	3.92x10-15	18.3	PC
rs4579515	8:59476807	A	G	*AC068522.4*	ENSG00000242970	cis	3.73x10-15	18.3	PPG
rs72805116	16:85792967	A	G	*C16orf74*	ENSG00000154102	cis	3.57x10-15	18.3	lncRNA
rs112697442	14:35580186	C	T	*SEPT7P1*	ENSG00000259090	cis	4.51x10-15	18.2	PPG
rs1411476	1:104569468	C	T	*RP11.364B6.2*	ENSG00000215869	cis	5.64x10-15	18.1	PC
rs142863425	6:27885980	C	T	*ZNF389*	ENSG00000226314	cis	6.07x10-15	18.1	PC
rs17216887	4:100010273	C	G	*RP11.696N14.1*	ENSG00000246090	cis	5.73x10-15	18.1	PC
rs2269984	7:141362693	A	T	*RP5.894A10.6*	ENSG00000270157	cis	5.46x10-15	18.1	PC
rs2314645	4:15538922	G	T	*ADRB3*	ENSG00000188778	trans	5.99x10-15	18.1	PC
rs2315601	17:39086932	C	T	*KRT23*	ENSG00000108244	cis	6.04x10-15	18.1	TCPPG
rs2739337	22:24296483	C	T	*DDT*	ENSG00000099977	cis	6.23x10-15	18.1	TCunPPG
rs2831947	21:30022213	A	G	*CCT8*	ENSG00000156261	cis	5.57x10-15	18.1	lncRNA
rs28565512	4:57397196	C	G	*THEGL*	ENSG00000249693	cis	6.34x10-15	18.1	PC
rs797515	13:51227415	G	A	*DLEU7*	ENSG00000186047	cis	5.67x10-15	18.1	lncRNA
rs113734006	8:59345027	C	T	*UBXN2B*	ENSG00000215114	cis	7.6x10-15	18	PC
rs1991401	17:62502435	A	G	*DDX5*	ENSG00000108654	cis	6.94x10-15	18	PC
rs4273875	8:2585105	T	C	*GS1.57L11.1*	ENSG00000253853	cis	6.5x10-15	18	PC
rs77245739	16:4801939	G	T	*ZNF500*	ENSG00000103199	cis	7.53x10-15	18	PC
rs78095701	17:66443609	A	C	*WIPI1*	ENSG00000070540	cis	6.42x10-15	18	lncRNA
rs112521249	14:35244362	C	T	*RP11.73E17.2*	ENSG00000258738	cis	8.3x10-15	17.9	PC
rs11631833	15:75588262	G	A	*CTD.2323K18.1*	ENSG00000260269	cis	9.14x10-15	17.9	PC
rs11668526	19:54749060	C	T	*LILRA6*	ENSG00000244482	cis	8.6x10-15	17.9	PC
rs4141794	7:130667258	G	A	*AMER2*	ENSG00000165566	trans	7.86x10-15	17.9	PC
rs4687658	3:52860366	C	G	*RP5.966M1.6*	ENSG00000243696	cis	8.27x10-15	17.9	TCunPPG
rs4804785	19:7780643	A	C	*CLEC4G*	ENSG00000182566	cis	7.95x10-15	17.9	PPG
rs56053835	15:85093046	G	A	*UBE2Q2P1*	ENSG00000189136	cis	8.47x10-15	17.9	PC

eQTL rs ID	eQTL chr:pos	ref	alt	Gene symbol	Ensembl gene ID	cis/trans	eQTL P-value	R^2	Gene type
rs708277	16:54147935	G	A	*FTO*	ENSG00000140718	cis	9.17x10-15	17.9	PC
rs78622635	1:16538094	A	G	*ARHGEF19*	ENSG00000142632	cis	8.34x10-15	17.9	lncRNA
rs9842198	3:156539204	G	C	*PA2G4P4*	ENSG00000230457	cis	8.35x10-15	17.9	lncRNA
rs10151105	14:62549004	G	A	*RP11.355I22.3*	ENSG00000186369	cis	1.14x10-14	17.8	PC
rs11653833	17:15592143	C	G	*ZNF286B*	ENSG00000249459	trans	1.07x10-14	17.8	PC
rs12927623	16:34021783	A	G	*DUSP22*	ENSG00000112679	trans	1.05x10-14	17.8	TCunitary PG
rs2712204	7:107148544	A	T	*GPR22*	ENSG00000172209	cis	1.14x10-14	17.8	NA
rs2985424	1:145386391	A	G	*RP11.458D21.5*	ENSG00000213240	cis	1.04x10-14	17.8	lncRNA
rs34352889	10:88974706	G	A	*RP11.506M13.3*	ENSG00000244733	trans	1.11x10-14	17.8	TCunPPG
rs11118912	1:222149130	C	T	*RP11.815M8.1*	ENSG00000238042	cis	1.42x10-14	17.7	PC
rs11743541	5:75738858	G	A	*CTD.2236F14.1*	ENSG00000249713	cis	1.39x10-14	17.7	PC
rs2454705	12:123192848	G	A	*RP11.324E6.6*	ENSG00000256249	cis	1.28x10-14	17.7	lncRNA
rs2961559	10:4864809	T	C	*AKR1E2*	ENSG00000165568	cis	1.39x10-14	17.7	PC
rs35775224	7:7015632	A	C	*RP11.266K4.12*	ENSG00000256589	trans	1.27x10-14	17.7	lncRNA
rs56288025	19:58963717	G	T	*ZNF324B*	ENSG00000249471	cis	1.19x10-14	17.7	lncRNA
rs60163605	17:43334649	C	A	*SPATA32*	ENSG00000184361	cis	1.3x10-14	17.7	unPPG
rs1227809	19:37269981	G	C	*ZNF790*	ENSG00000197863	cis	1.51x10-14	17.6	PC
rs12814509	12:21613367	A	T	*PYROXD1*	ENSG00000121350	cis	1.47x10-14	17.6	PC
rs200210467	14:59983185	T	G	*CCDC175*	ENSG00000151838	cis	1.79x10-14	17.6	PC
rs2904758	6:29923351	G	A	*HLA.W*	ENSG00000235290	cis	1.46x10-14	17.6	lncRNA
rs62147427	2:87126664	C	T	*RP1.81D8.3*	ENSG00000224477	trans	1.46x10-14	17.6	unPPG
rs1803005	6:24804860	G	T	*C6orf229*	ENSG00000260286	cis	1.82x10-14	17.5	PC
rs2271089	17:79210707	G	A	*C17orf89*	ENSG00000224877	cis	2.47x10-14	17.4	PC
rs2942202	8:23418444	A	C	*PRDM6*	ENSG00000061455	trans	2.31x10-14	17.4	PC
rs76021834	7:75607155	A	G	*MDH2*	ENSG00000146701	cis	2.26x10-14	17.4	PC
rs10081693	9:94966486	T	G	*ASPN*	ENSG00000106819	cis	2.89x10-14	17.3	PC
rs10255707	7:45954692	T	C	*AC073115.7*	ENSG00000229628	cis	2.8x10-14	17.3	PC
rs12168582	22:28316227	A	G	*TTC28*	ENSG00000100154	cis	2.71x10-14	17.3	PC
rs1883595	6:40935728	G	A	*APOBEC2*	ENSG00000124701	cis	2.86x10-14	17.3	PC
rs251732	16:4310468	C	G	*TFAP4*	ENSG00000090447	cis	2.81x10-14	17.3	lncRNA
rs3807891	7:95226709	G	C	*AC002451.3*	ENSG00000231170	cis	3.19x10-14	17.3	lncRNA
rs62065454	17:43573592	A	G	*RP11.156P1.1*	ENSG00000263142	trans	2.86x10-14	17.3	TCunPPG
rs12453507	17:38053207	C	G	*GSDMB*	ENSG00000073605	cis	3.96x10-14	17.2	PC
rs147751661	17:62927969	G	A	*RP11.197O15.1*	ENSG00000214401	trans	3.73x10-14	17.2	PC
rs4754708	11:100759034	A	G	*ARHGAP42*	ENSG00000165895	cis	3.34x10-14	17.2	lncRNA
rs12539729	7:128142023	A	T	*METTL2B*	ENSG00000165055	cis	4.3x10-14	17.1	PC
rs4647870	17:74516655	A	C	*SNHG16*	ENSG00000163597	cis	4.25x10-14	17.1	lncRNA
rs4736693	8:134553603	G	C	*ERICH3*	ENSG00000178965	trans	4.79x10-14	17.1	PC
rs58173607	18:74277779	G	T	*RP11.111H3.2*	ENSG00000266256	cis	4.99x10-14	17.1	PC
rs60659886	1:26994245	A	T	*PIGV*	ENSG00000060642	cis	4.33x10-14	17.1	PC
rs7082545	10:52478100	T	G	*ASAH2B*	ENSG00000204147	cis	4.11x10-14	17.1	lncRNA
rs73359005	8:135795757	G	A	*AC083843.1*	ENSG00000259820	cis	4.85x10-14	17.1	lncRNA
rs11042607	11:2121817	A	G	*IGF2*	ENSG00000167244	cis	5.15x10-14	17	PC
rs117491574	17:58325048	T	G	*RP11.3K24.1*	ENSG00000241157	cis	5.02x10-14	17	PC

eQTL rs ID	eQTL chr:pos	ref	alt	Gene symbol	Ensembl gene ID	cis/trans	eQTL P-value	R^2	Gene type
rs11951414	5:68431872	C	T	*RP11.309M7.1*	ENSG00000261298	trans	5.85x10-14	17	PC
rs17259058	7:87921277	C	G	*AC003991.3*	ENSG00000228113	cis	5.72x10-14	17	PC
rs2230707	1:19202896	G	A	*ALDH4A1*	ENSG00000159423	cis	5.13x10-14	17	lncRNA
rs34630775	8:79417693	G	T	*RP11.578O24.2*	ENSG00000254352	cis	5.17x10-14	17	PPG
rs71473137	15:30948494	C	T	*RP11.932O9.9*	ENSG00000269930	cis	5.45x10-14	17	PPG
rs72751993	1:233468388	T	C	*RP5.862P8.2*	ENSG00000143674	cis	5.21x10-14	17	lncRNA
rs867845230	17:62920612	A	G	*RP11.583F2.1*	ENSG00000264057	cis	6.09x10-14	17	TCunPPG
rs9303577	17:49249186	C	A	*NME1.NME2*	ENSG00000011052	cis	6.08x10-14	17	lncRNA
rs1665294	2:114369935	T	G	*AC016629.3*	ENSG00000269600	trans	6.24x10-14	16.9	PC
rs61978213	14:70653758	G	A	*SLC8A3*	ENSG00000100678	cis	6.89x10-14	16.9	lncRNA
rs12149595	16:35259917	G	A	*DUSP22*	ENSG00000112679	trans	9.01x10-14	16.8	PC
rs3115858	1:755890	A	T	*FAM87B*	ENSG00000177757	cis	7.68x10-14	16.8	PC
rs6503453	17:44062603	A	G	*AC007679.3*	ENSG00000225610	trans	9.16x10-14	16.8	lncRNA
rs6866645	5:137988944	A	G	*LRRTM2*	ENSG00000146006	cis	7.63x10-14	16.8	lncRNA
rs1852270	17:43655280	G	T	*LRRC37A3*	ENSG00000176809	trans	1.02x10-13	16.7	PC
rs28709161	22:22646562	T	C	*RP11.23E10.3*	ENSG00000260626	trans	9.88x10-14	16.7	lncRNA
rs7446704	5:70662060	C	A	*RP11.1280N14.4*	ENSG00000215630	cis	9.27x10-14	16.7	unPPG
rs10907576	9:46918872	A	G	*RP11.262H14.3*	ENSG00000234665	trans	1.14x10-13	16.6	lncRNA
rs12603589	17:65825248	T	C	*AC005332.1*	ENSG00000237854	cis	1.15x10-13	16.6	PC
rs13122	14:31359024	A	T	*RP11.829H16.3*	ENSG00000258525	cis	1.17x10-13	16.6	PC
rs3016125	22:20475050	T	C	*TMEM191C*	ENSG00000206140	trans	1.15x10-13	16.6	PC
rs3106149	17:62919455	G	A	*RP11.798G7.3*	ENSG00000214425	trans	1.3x10-13	16.6	TCunPPG
rs62307246	4:70295728	A	C	*UGT2B11*	ENSG00000213759	cis	1.19x10-13	16.6	lncRNA
rs62542381	9:27488566	C	T	*RP11.120I21.2*	ENSG00000254202	trans	1.13x10-13	16.6	TCunPPG
rs75655792	7:99813285	C	A	*PVRIG*	ENSG00000213413	cis	1.14x10-13	16.6	lncRNA
rs184608345	9:66584918	C	T	*RP11.745L13.2*	ENSG00000251555	trans	1.42x10-13	16.5	lncRNA
rs185073	5:179334857	A	G	*TBC1D9B*	ENSG00000197226	cis	1.61x10-13	16.5	PC
rs2562240	16:83458	A	G	*AP006222.1*	ENSG00000228463	trans	1.63x10-13	16.5	PC
rs28551168	1:229456185	C	T	*CCSAP*	ENSG00000154429	cis	1.5x10-13	16.5	PC
rs4356723	20:24899051	T	C	*ACSS1*	ENSG00000154930	cis	1.63x10-13	16.5	PC
rs56248825	21:39465015	C	T	*SCAMP4*	ENSG00000227500	trans	1.54x10-13	16.5	PC
rs59812768	1:198599981	T	C	*RP11.55L3.1*	ENSG00000250791	trans	1.59x10-13	16.5	TCPPG
rs77913677	3:122038460	T	C	*KPNA1*	ENSG00000114030	cis	1.52x10-13	16.5	lncRNA
rs113352051	2:89283617	T	C	*IGKV1.9*	ENSG00000241755	cis	1.69x10-13	16.4	lncRNA
rs148240181	12:93080	C	G	*ABC7.42389800N19.1*	ENSG00000226210	cis	1.9x10-13	16.4	PC
rs1641716	12:12224718	G	A	*BCL2L14*	ENSG00000121380	cis	1.84x10-13	16.4	unPPG
rs4798075	18:3247256	A	C	*RP13.270P17.3*	ENSG00000272688	cis	2x10-13	16.4	IG V gene
rs62253656	3:53023428	G	C	*RP11.894J14.5*	ENSG00000272305	cis	1.84x10-13	16.4	PC
rs1035559	16:72031860	A	G	*DHX38*	ENSG00000140829	cis	2.43x10-13	16.3	lncRNA
rs1046381	1:153604112	G	A	*RP1.178F15.5*	ENSG00000271853	cis	2.34x10-13	16.3	PC
rs13063033	3:46082481	G	A	*RP11.793E15.1*	ENSG00000229515	cis	2.47x10-13	16.3	PC
rs2396690	6:46618289	C	G	*TDRD6*	ENSG00000180113	cis	2.26x10-13	16.3	PC
rs2644720	11:10859439	C	T	*SNORD97*	ENSG00000238622	cis	2.12x10-13	16.3	PC
rs372107064	5:21303103	G	C	*RP11.457M11.5*	ENSG00000261584	trans	2.15x10-13	16.3	PPG
rs55699816	3:196059377	A	T	*TCTEX1D2*	ENSG00000213123	cis	2.36x10-13	16.3	snoRNA

eQTL rs ID	eQTL chr:pos	ref	alt	Gene symbol	Ensembl gene ID	cis/trans	eQTL P-value	R^2	Gene type
rs6718328	2:198038562	G	A	*PPP2R2C*	ENSG00000074211	trans	2.28x10-13	16.3	lncRNA
rs1784304	11:118962816	C	A	*HMBS*	ENSG00000256269	cis	2.69x10-13	16.2	lncRNA
rs4542712	17:7469327	T	C	*AC113189.5*	ENSG00000233223	cis	2.67x10-13	16.2	PC
rs67654984	8:135707982	G	A	*CTD.2342N23.3*	ENSG00000272456	cis	2.54x10-13	16.2	PPG
rs7145650	14:35947295	G	A	*RP11.287A8.2*	ENSG00000229419	trans	2.69x10-13	16.2	lncRNA
rs7537908	1:78518939	A	G	*GIPC2*	ENSG00000137960	cis	2.81x10-13	16.2	PPG
rs80284522	3:139035188	T	C	*RP11.319G6.1*	ENSG00000248932	cis	2.74x10-13	16.2	lncRNA
rs869738	2:64493422	C	G	*AC114752.2*	ENSG00000236148	cis	2.8x10-13	16.2	PC
rs10025792	4:164504947	T	G	*RP11.557L19.1*	ENSG00000272002	trans	3.15x10-13	16.1	lncRNA
rs11027831	11:3550720	C	T	*CTC.342M10.2*	ENSG00000255367	cis	3.15x10-13	16.1	PC
rs11186897	10:94048558	C	G	*Mar-05*	ENSG00000198060	cis	3.08x10-13	16.1	PC
rs1817104	12:11338589	G	T	*TAS2R63P*	ENSG00000256019	cis	3.3x10-13	16.1	PC
rs200601667	2:107117022	A	AATTT	*AC108868.4*	ENSG00000254126	cis	3.5x10-13	16.1	PC
rs4782394	16:88726726	T	G	*RP5.1142A6.3*	ENSG00000260630	cis	3.46x10-13	16.1	PC
rs5744709	5:74891002	G	A	*ANKDD1B*	ENSG00000189045	cis	3.64x10-13	16.1	PPG
rs62461484	7:63911740	T	C	*ERV3.1*	ENSG00000213462	cis	3.14x10-13	16.1	PC
rs6737901	2:169936393	A	G	*DHRS9*	ENSG00000073737	cis	3.71x10-13	16.1	lncRNA
rs76330394	7:102791112	A	C	*RP11.401L13.4*	ENSG00000224415	cis	3.21x10-13	16.1	unPPG
rs796704680	15:32838699	T	C	*GOLGA8R*	ENSG00000186399	trans	3.19x10-13	16.1	lncRNA
rs13238996	7:74069645	A	G	*GTF2IRD2B*	ENSG00000174428	cis	4.44x10-13	16	lncRNA
rs17692129	17:44793283	C	T	*RP11.927P21.12*	ENSG00000271974	trans	3.87x10-13	16	PC
rs530569	11:94169708	C	T	*MIR548A1*	ENSG00000207775	trans	3.88x10-13	16	miRNA
rs142893200	15:84897901	A	G	*GOLGA6L9*	ENSG00000197978	trans	5.41x10-13	15.9	TCPPG
rs61842918	10:47746978	C	A	*ANXA8*	ENSG00000265190	cis	5.59x10-13	15.9	PC
rs62118921	19:23798289	C	T	*ZNF681*	ENSG00000196172	cis	5.24x10-13	15.9	PC
rs184608345	9:66584918	C	T	*RP11.204M4.2*	ENSG00000204837	trans	6.15x10-13	15.8	PC
rs188871467	16:8928935	T	G	*RP11.77H9.2*	ENSG00000260276	cis	5.68x10-13	15.8	PC
rs6770645	3:40227789	G	A	*MYRIP*	ENSG00000170011	cis	6.02x10-13	15.8	PC
rs7399685	13:114516947	A	G	*TMEM255B*	ENSG00000184497	cis	6.17x10-13	15.8	TCunPPG
rs11123747	2:99328763	T	C	*GABRB2*	ENSG00000145864	trans	7.04x10-13	15.7	lncRNA
rs142893200	15:84897901	A	G	*RP11.152F13.3*	ENSG00000255769	trans	8.07x10-13	15.7	PC
rs146957881	5:177233117	C	A	*RP11.1277A3.2*	ENSG00000246596	cis	7.75x10-13	15.7	PC
rs200690889	9:44544142	G	A	*RP11.111F5.5*	ENSG00000227449	trans	8.27x10-13	15.7	TCunPPG
rs202086943	20:29602222	T	G	*MLLT10P1*	ENSG00000238151	cis	7.66x10-13	15.7	TCunPPG
rs4476463	3:36880858	A	G	*AC011816.1*	ENSG00000234073	cis	7.43x10-13	15.7	PPG
rs57947330	17:16728955	T	C	*CCDC144A*	ENSG00000170160	cis	7.87x10-13	15.7	PPG
rs57947330	17:16728955	T	C	*USP32P1*	ENSG00000188933	cis	7.53x10-13	15.7	TCunPPG
rs61998439	15:31111123	G	T	*RP11.540B6.6*	ENSG00000270015	cis	7.72x10-13	15.7	NA
rs74651789	4:165898682	C	T	*RP11.366M4.11*	ENSG00000248632	cis	7.4x10-13	15.7	TCunPPG
rs11052241	12:9811948	A	G	*RP3.337H4.8*	ENSG00000203362	trans	8.33x10-13	15.6	NA
rs17216887	4:100010273	C	G	*ADH6*	ENSG00000172955	cis	8.98x10-13	15.6	PC
rs3783164	13:41380363	C	T	*RP11.346L13.3*	ENSG00000168852	cis	8.68x10-13	15.6	PC
rs4242389	8:22972321	G	A	*POU5F1B*	ENSG00000212993	trans	9.81x10-13	15.6	PC
rs56065326	3:112008771	C	T	*SLC9C1*	ENSG00000172139	cis	8.44x10-13	15.6	TCunPPG
rs56185013	4:106160133	G	A	*TET2*	ENSG00000168769	cis	9.51x10-13	15.6	PC

eQTL rs ID	eQTL chr:pos	ref	alt	Gene symbol	Ensembl gene ID	cis/trans	eQTL P-value	R^2	Gene type
rs61809245	1:148203960	G	A	*NBPF20*	ENSG00000162825	trans	8.88x10-13	15.6	PC
rs62364993	5:80514325	G	C	*CKMT2*	ENSG00000131730	cis	8.58x10-13	15.6	PC
rs6488334	12:11140444	T	C	*TAS2R30*	ENSG00000256188	cis	9.02x10-13	15.6	lncRNA
rs72905231	2:191303525	A	G	*NEMP2*	ENSG00000189362	cis	9.05x10-13	15.6	PC
rs77391378	21:29947914	T	A	*AF131217.1*	ENSG00000232855	cis	9.87x10-13	15.6	lncRNA
rs776296	7:63804337	T	A	*ZNF736*	ENSG00000234444	cis	8.84x10-13	15.6	PC
rs1233578	6:28712247	A	G	*BTN3A2*	ENSG00000186470	trans	1.14x10-12	15.5	PC
rs2697291	2:198111312	A	G	*RP11.794P6.1*	ENSG00000255428	trans	1.12x10-12	15.5	PC
rs61979250	14:76668905	C	G	*GPATCH2L*	ENSG00000089916	cis	1.09x10-12	15.5	PC
rs34019730	16:89812930	G	A	*FANCA*	ENSG00000187741	cis	1.24x10-12	15.4	lncRNA
rs35084227	2:87277202	T	C	*PLG*	ENSG00000122194	trans	1.25x10-12	15.4	PC
rs35785032	4:175697250	G	A	*ADAM29*	ENSG00000168594	cis	1.49x10-12	15.4	PC
rs372161775	5:175398456	A	G	*THOC3*	ENSG00000051596	cis	1.29x10-12	15.4	PC
rs41265179	1:153601828	G	T	*NUP210L*	ENSG00000143552	cis	1.4x10-12	15.4	PC
rs10584	15:90328551	G	C	*ANPEP*	ENSG00000166825	cis	1.72x10-12	15.3	PC
rs11246329	11:843713	C	T	*TSPAN4*	ENSG00000214063	cis	1.72x10-12	15.3	PC
rs12812329	12:9167788	T	C	*RP11.436I9.3*	ENSG00000255776	cis	1.54x10-12	15.3	PC
rs2684556	17:44580533	A	G	*RP11.156P1.1*	ENSG00000263142	cis	1.56x10-12	15.3	PC
rs3807020	6:41747048	G	C	*OR56A1*	ENSG00000180934	trans	1.62x10-12	15.3	PC
rs56175172	11:77245728	A	G	*AP000580.1*	ENSG00000219529	cis	1.51x10-12	15.3	PPG
rs61945858	12:120879461	A	G	*DYNLL1*	ENSG00000088986	cis	1.62x10-12	15.3	PPG
rs62414240	6:76274825	C	T	*RNU6.1016P*	ENSG00000252498	cis	1.72x10-12	15.3	snRNA
rs7448600	5:21365623	C	A	*RP11.823P9.3*	ENSG00000233974	cis	1.82x10-12	15.3	PPG
rs10435105	7:3330994	C	T	*SDK1*	ENSG00000146555	cis	1.97x10-12	15.2	TCunPPG
rs112161250	15:58440089	G	GCA	*RP1*	ENSG00000104237	trans	1.94x10-12	15.2	PC
rs9567377	13:44720217	G	T	*SMIM2.AS1*	ENSG00000227258	cis	2x10-12	15.2	PC

Supplementary Table 3 Phenotypes from the NHGRI-EBI GWAS Catalog of published genome-wide association studies reported for novel and confirmed eQTL.

The full list of phenotypes reported for eQTL in the PAH Cohort study including sixty-three eQTL reported for at least one lung-related phenotype in the GWAS Catalog are listed in the column 'GWAS Catalog phenotypes'. The column 'confirmation' indicates if the eQTL was found in the population-based studies used for confirmation.

eQTL rs ID	Gene symbol	confirmation	GWAS Catalog phenotypes
rs10790962	*SEPT3*	novel	Glaucoma; Glaucoma (multi-trait analysis); Intraocular pressure; Lung function (FVC); Psoriasis; Multiple sclerosis; Psoriasis vulgaris; Celiac disease; Lymphocyte percentage of white cells
rs7947900	*IFITM2*	novel	Eosinophil counts; Red blood cell count; Lung function (FEV1/FVC); White blood cell count; Granulocyte percentage of myeloid white cells; Monocyte percentage of white cells; Monocyte count; Neutrophil percentage of white cells; Lymphocyte percentage of white cells; Myeloid white cell count; White blood cell count (basophil); Sum neutrophil eosinophil counts; Granulocyte count; Neutrophil count; Sum basophil neutrophil counts; Medication use (thyroid preparations)
rs1451659	*OR51R1P*	novel	Lung cancer
rs2008345	*RP11.298I3.1*	novel	Lung function (FEV1/FVC); Household income (MTAG); Cognitive ability; years of educational attainment or schizophrenia (pleiotropy); Cognitive ability (MTAG); Educational attainment (years of education); Educational attainment (MTAG); Highest math class taken; Cognitive performance (MTAG); Self-reported math ability; Highest math class taken (MTAG)
rs10136164	*PAX9*	novel	Lung function (FVC)
rs72785089	*CLEC18A*	novel	Lung function (FVC)
rs8058597	*NPIPB15*	novel	Lung function (FVC)
rs111164082	*RP11.798G7.3*	novel	Interstitial lung disease; Progressive supranuclear palsy; Risk-taking tendency (4-domain principal component model); Depressed affect; Eosinophil counts; Worry; Neuroticism; Number of sexual partners; Red blood cell count; General cognitive ability; Lung function (FVC); Male-pattern baldness; Alcohol consumption; Ease of getting up in the morning; Daytime nap; White matter microstructure (fractional anisotropy); Parkinson's disease; Alcohol use disorder (total score); Alcohol use disorder (consumption score); Osteoarthritis (hip); Sleep duration; Automobile speeding propensity; Brain region volumes; General factor of neuroticism; Primary biliary cirrhosis; Neurociticism; Irritable mood; Feeling guilty; Feeling miserable; Experiencing mood swings; Worry too long after an embarrassing experience; Feeling hurt; Feeling worry; Type 1 diabetes; Waist-hip ratio
rs3785884	*LRRC37A2*	novel	Interstitial lung disease; Progressive supranuclear palsy; Risk-taking tendency (4-domain principal component model); Depressed affect; Eosinophil counts; Worry; Neuroticism; Number of sexual partners; Red blood cell count; General cognitive ability; Lung function (FVC); Household income; Male-pattern baldness; Alcohol consumption; Ease of getting up in the morning; Daytime nap; White matter microstructure (fractional anisotropy); Parkinson's disease; Alcohol use disorder (total score); Alcohol use disorder (consumption score); Osteoarthritis (hip); Sleep duration; Automobile speeding propensity; Brain region volumes; General factor of neuroticism; Neurociticism; Irritable mood; Feeling guilty; Feeling miserable; Experiencing mood swings; Worry

eQTL rs ID	Gene symbol	confirmation	GWAS Catalog phenotypes
			too long after an embarrassing experience; Feeling hurt; Feeling worry; Type 1 diabetes; Waist-hip ratio; Medication use (anilides)
rs112534607	*AC126544.4*	novel	Lung function in never smokers (low FEV1 vs high FEV1); Loneliness; White matter microstructure (radial diusivities); White matter microstructure (axial diusivities); White matter microstructure (mean diusivities); White matter microstructure (fractional anisotropy); General cognitive ability; Neuroticism; Feeling guilty; Feeling miserable; Experiencing mood swings; Red cell distribution width; Mood instability; Breast cancer
rs2732617	*RP11.359G22.2*	novel	Lung function in never smokers (low FEV1 vs high FEV1); Loneliness; White matter microstructure (radial diusivities); White matter microstructure (axial diusivities); White matter microstructure (mean diusivities); White matter microstructure (fractional anisotropy); General cognitive ability; Neuroticism; Feeling guilty; Feeling miserable; Experiencing mood swings; Red cell distribution width; Mood instability; Breast cancer
rs2732603	*RP11.583F2.1*	novel	Lung function in never smokers (low FEV1 vs high FEV1); Loneliness; White matter microstructure (radial diusivities); White matter microstructure (axial diusivities); White matter microstructure (mean diusivities); White matter microstructure (fractional anisotropy); General cognitive ability; Neuroticism; Feeling guilty; Feeling miserable; Experiencing mood swings; Red cell distribution width; Mood instability; Breast cancer
rs8073963	*HOXB.AS1*	novel	Lung function (FEV1/FVC); Primary tooth development (number of teeth)
rs35858034	*CYP2T2P*	novel	Smoking behavior; Chronic obstructive pulmonary disease; Smoking behaviour (cigarettes smoked per day); Blood protein levels; Post bronchodilator FEV1/FVC ratio; Emphysema imaging phenotypes; Post bronchodilator FEV1; Hemoglobin concentration
rs2353408	*U2SURP*	novel	Red cell distribution width; Lung function (FEV1/FVC); Urate levels
rs9842198	*PA2G4P4*	novel	Sunburns; Interleukin-2 levels; Nose size; Breast cancer; Chronic obstructive pulmonary disease or resting heart rate (pleiotropy)
rs10079553	*RUFY1*	novel	Lung cancer
rs6925979	*ADGRG6*	novel	Height; Scoliosis; Body fat distribution (trunk fat ratio); Body fat distribution (leg fat ratio); Body fat distribution (arm fat ratio); Heel bone mineral density; Chronic obstructive pulmonary disease; Adolescent idiopathic scoliosis; Waist circumference adjusted for body mass index; Waist circumference adjusted for BMI in non-smokers; Waist circumference adjusted for BMI in smokers; Waist circumference adjusted for BMI (adjusted for smoking behaviour); Waist circumference adjusted for BMI (joint analysis main effects and smoking interaction); Chronic obstructive pulmonary disease or high blood pressure (pleiotropy)
rs2769345	*RP11.514O12.4*	novel	Crohn's disease; Vitiligo; Autoimmune traits; Hypothyroidism; Graves' disease; Inflammatory bowel disease; Lung cancer; Lung adenocarcinoma; Basal cell carcinoma; Medication use (thyroid preparations)
rs9272779	*HLA.DQA1*	novel	Schizophrenia; Lung function in never smokers (low FEV1 vs high FEV1); Self-reported allergy; Composite immunoglobulin trait (IgA x IgG x IgM); Type 2 diabetes; Childhood steroid-sensitive nephrotic syndrome; Asthma and hay fever; Allergic sensitization; Epstein Barr virus nuclear antigen 1 IgG levels; Epstein Barr virus nuclear antigen 1 IgG seropositivity; Epstein-Barr virus immune response (multivariate analysis); Autism spectrum disorder or schizophrenia; Asthma or allergic disease (pleiotropy); Asthma; Hypothyroidism; Ulcerative colitis
rs9273495	*HLA.DQB2*	novel	Asthma; Ulcerative colitis; Chronic lymphocytic leukemia; Type 1 diabetes; Type 2 diabetes; Eosinophilic granulomatosis with polyangiitis; Asthma and hay fever; Epstein Barr virus nuclear antigen 1 IgG levels; Asthma or allergic disease (pleiotropy); Blood protein levels; Lung function (FEV1); Lung function (FEV1/FVC); Inflammatory

eQTL rs ID	Gene symbol	confirmation	GWAS Catalog phenotypes
			bowel disease; Medication use (drugs used in diabetes)
rs9273841	*XXbac.BPG254F23.5*	novel	Immunoglobulin A; Multiple sclerosis; Ulcerative colitis or Crohn's disease; Systemic lupus erythematosus; Lymphoma; Ulcerative colitis; Multiple sclerosis (OCB status); Appendicular lean mass; FEV1; Lung function (FEV1/FVC); Peak expiratory flow; Follicular lymphoma; Hypothyroidism; Blood protein levels; Hepatitis C (spontaneous viral clearance); Random C-peptide levels in type I diabetes; C-peptide levels in type I diabetes; Hepatocellular carcinoma in hepatitis C infection; Urinary tract infection frequency; Hepatitis B; Itch intensity from mosquito bite adjusted by bite size; Mosquito bite size; Perceived unattractiveness to mosquitoes; Itch intensity from mosquito bite; Crohn's disease; IgA nephropathy; Lupus nephritis in systemic lupus erythematosus; C-reactive protein levels or total cholesterol levels (pleiotropy); Oral cavity and pharyngeal cancer; Oropharynx cancer; Oral cavity cancer
rs13238996	*GTF2IRD2B*	novel	Red cell distribution width; Cardiovascular disease; Lung function (FEV1/FVC); Body mass index; Menarche (age at onset); Chronic obstructive pulmonary disease or high blood pressure (pleiotropy); Diastolic blood pressure
rs7543453	*RP11.305E17.6*	confirmed	Lung cancer in ever smokers
rs760077	*THBS3*	confirmed	Renal function-related traits (BUN); Magnesium levels; Esophageal cancer and gastric cancer; Urate levels; Occipital lobe volume; General risk tolerance (MTAG); Red blood cell count; Serum urea levels; Lung function (FVC); Serum cancer antigen 15.3 levels; PR interval; Diastolic blood pressure; Mean arterial pressure; Systolic blood pressure; Dentures; Brain region volumes; Heel bone mineral density; Number of decayed; missing and filled tooth surfaces or use of dentures; Gastric cancer; Serum uric acid levels; Blood urea nitrogen levels; Estimated glomerular filtration rate; Non-cardia gastric cancer; Urinary albumin-to-creatinine ratio in non-diabetics; Urinary albumin-to-creatinine ratio; Gastric adenocarcinoma (histologically verified); Cancer (pleiotropy); Cancer; Hematocrit; Chronic inflammatory diseases (ankylosing spondylitis; Crohn's disease; psoriasis; primary sclerosing cholangitis; ulcerative colitis) (pleiotropy); Hemoglobin concentration; Urea levels; Serum magnesium levels; Gout; Microalbuminuria
rs6480781	*C10orf11*	confirmed	Lung function (FEV1/FVC)
rs2342607	*TMEM254.AS1*	confirmed	Chronic obstructive pulmonary disease-related biomarkers
rs2368238	*MPZL2*	confirmed	Blood protein levels; Lung adenocarcinoma; Non-small cell lung cancer; Lung cancer
rs11231152	*RP11.371A22.1*	confirmed	Waist-hip ratio; Heel bone mineral density; Waist-to-hip ratio adjusted for BMI; Lung function (FVC); FEV1; Peak expiratory flow; Blood protein levels; Waist-to-hip ratio adjusted for body mass index; Lung function (FEV1); Waist-to-hip ratio adjusted for BMI (joint analysis for main effect and physical activity interaction); Waist-to-hip ratio adjusted for BMI in active individuals; Waist-to-hip ratio adjusted for BMI x sex x age interaction (4df test)
rs61705659	*ARHGEF17*	confirmed	Pulmonary function (smoking interaction); FEV1; Lung function (FEV1/FVC); Peak expiratory flow; GIP levels in response to oral glucose tolerance test (120 minutes)
rs10876864	*RPS26*	confirmed	Type 1 diabetes; Vitiligo; Polycystic ovary syndrome; Appendicular lean mass; Smoking status (ever vs never smokers); Eosinophil counts; Red blood cell count; Respiratory diseases; Autoimmune traits; General cognitive ability; Lung function (FEV1/FVC); FEV1; Asthma; Eczema; Hypothyroidism; Menarche (age at onset); Hay fever and/or eczema; Household income (MTAG); Nasal polyps; Cognitive ability; years of educational attainment or schizophrenia (pleiotropy); Smoking status; Inflammatory skin disease; Alopecia areata; Lung function (FEV1);

eQTL rs ID	Gene symbol	confirmation	GWAS Catalog phenotypes
			Body mass index; Rheumatoid arthritis; Heel bone mineral density; Age of smoking initiation (MTAG); Asthma (childhood onset); Intelligence (MTAG); Cognitive ability (MTAG); Educational attainment (years of education); Smoking initiation (ever regular vs never regular); Gastroesophageal reflux disease; Asthma or allergic disease (pleiotropy); Pediatric autoimmune diseases; Nonsyndromic cleft lip with cleft palate; Eosinophil percentage of white cells; Educational attainment (college completion); Eosinophil percentage of granulocytes; Sum eosinophil basophil counts; Neutrophil percentage of granulocytes; Allergic disease (asthma; hay fever or eczema); Asthma (adult onset); Medication use (drugs for peptic ulcer and gastro-oesophageal reflux disease); Medication use (glucocorticoids); Medication use (adrenergics; inhalants); Medication use (thyroid preparations); Asthma (moderate or severe); Educational attainment (MTAG); Self-reported math ability (MTAG); Highest math class taken; Cognitive performance; Cognitive performance (MTAG)
rs1131017	*AC004057.1*	confirmed	Type 1 diabetes; Vitiligo; Polycystic ovary syndrome; Appendicular lean mass; Smoking status (ever vs never smokers); Red blood cell count; Respiratory diseases; General cognitive ability; Lung function (FEV1/FVC); FEV1; Asthma; Eczema; Hypothyroidism; Menarche (age at onset); Hay fever and/or eczema; Allergic rhinitis; Household income (MTAG); Nasal polyps; Cognitive ability; years of educational attainment or schizophrenia (pleiotropy); Smoking status; Inflammatory skin disease; Alopecia areata; Lung function (FEV1); Body mass index; Rheumatoid arthritis; Heel bone mineral density; Brain region volumes; Lifetime smoking index; Age of smoking initiation (MTAG); Smoking initiation (ever regular vs never regular); Asthma (childhood onset); Cognitive function; Intelligence (MTAG); Cognitive ability (MTAG); Anorexia nervosa; Educational attainment (years of education); Smoking initiation; Allergic disease (asthma; hay fever or eczema); Asthma or allergic disease (pleiotropy); Pediatric autoimmune diseases; Eosinophil counts; Nonsyndromic cleft lip with cleft palate; Eosinophil percentage of white cells; Educational attainment (college completion); Eosinophil percentage of granulocytes; Neutrophil percentage of granulocytes; Asthma (adult onset); Smoking initiation (ever regular vs never regular) (MTAG); Medication use (glucocorticoids); Medication use (adrenergics; inhalants); Medication use (thyroid preparations); Asthma (moderate or severe); Educational attainment (MTAG); Self-reported math ability (MTAG); Highest math class taken; Cognitive performance; Cognitive performance (MTAG); Self-reported math ability; Highest math class taken (MTAG)
rs7147394	*HAUS4*	confirmed	Hypertension; Lung function (FEV1/FVC); Household income (MTAG); Cognitive ability; years of educational attainment or schizophrenia (pleiotropy); Cognitive ability (MTAG); Educational attainment (years of education); Educational attainment (MTAG); Highest math class taken; Cognitive performance (MTAG); Self-reported math ability; Highest math class taken (MTAG)
rs7183203	*FBN1*	confirmed	Breast cancer; Lung function (FEV1/FVC); Heel bone mineral density; Intraocular pressure
rs1628955	*ANKDD1A*	confirmed	Lung function (FEV1/FVC); Pulse pressure
rs34443712	*TSPAN3*	confirmed	Height; Red blood cell count; Lung function (FEV1/FVC)
rs2668714	*RP11.156P1.2*	confirmed	Lung function in never smokers (low FEV1 vs high FEV1); Loneliness; White matter microstructure (radial diusivities); White matter microstructure (axial diusivities); White matter microstructure (mean diusivities); White matter microstructure (fractional anisotropy); General cognitive ability; Neuroticism; Feeling guilty; Feeling miserable; Experiencing mood swings; Red cell distribution width; Mood instability; Breast cancer
rs2696506	*RP11.44K6.4*	confirmed	Lung function in never smokers (low FEV1 vs high FEV1); Loneliness; White matter microstructure (radial

eQTL rs ID	Gene symbol	confirmation	GWAS Catalog phenotypes
			diusivities); White matter microstructure (axial diusivities); White matter microstructure (mean diusivities); White matter microstructure (fractional anisotropy); General cognitive ability; Neuroticism; Feeling guilty; Feeling miserable; Experiencing mood swings; Red cell distribution width; Mood instability; Breast cancer
rs113680823	*DCXR*	confirmed	Lung function (FVC); FEV1
rs62113084	*ZFP82*	confirmed	Lung function (FVC)
rs406133	*SHKBP1*	confirmed	Age at loss of ambulation in Duchenne muscular dystrophy; Lung function (FVC); Smoking initiation (ever regular vs never regular) (MTAG)
rs4569473	*RP11.301O19.1*	confirmed	Lung function (FEV1/FVC); Pulse pressure; Diastolic blood pressure; Systolic blood pressure
rs2735313	*LCA5L*	confirmed	Lung function (FEV1/FVC); Platelet count
rs13051949	*WRB*	confirmed	Lung function (FEV1/FVC); Esotropia; Non-accommodative esotropia; Platelet count; Plateletcrit
rs1800818	*PDGFB*	confirmed	Lung function (FEV1/FVC)
rs6775611	*ANAPC13*	confirmed	Lung function (FVC); Height; Neurocognitive impairment in HIV-1 infection (dichotomous)
rs9852745	*RSRC1*	confirmed	Lung function in heavy smokers (high FEV1 vs average FEV1); Waist-hip ratio; Depression; Body mass index
rs11724788	*CYTL1*	confirmed	Lung function (FVC); Height
rs10064943	*RP11.43F13.1*	confirmed	Lung adenocarcinoma
rs12658646	*PDE8B*	confirmed	Caudate nucleus volume; Lung function (FEV1/FVC); Estimated glomerular filtration rate
rs4869923	*GINM1*	confirmed	Brainstem volume; Lung cancer
rs2393593	*HIST1H4H*	confirmed	Parental longevity (father's age at death or father's attained age); Offspring birth weight; Birth weight; Hand grip strength; Lifetime smoking index; Chronotype; Smoking cessation (MTAG); Lung cancer; Height; Cisplatin-induced ototoxicity; Educational attainment; Educational attainment (years of education); Educational attainment (MTAG)
rs67509210	*BTN3A2*	confirmed	Urinary metabolite modules (eigenmetabolites) in chronic kidney disease; General cognitive ability; Life satisfaction; Neuroticism; Hip circumference variance; Hip circumference; Intelligence (MTAG); Urinary tract infection frequency; Squamous cell lung carcinoma; Lung cancer in ever smokers; Headache; Urate levels
rs67859638	*BTN3A2*	confirmed	Urinary tract infection frequency; Squamous cell lung carcinoma
rs192804591	*HIST1H4L*	confirmed	Schizophrenia; White matter microstructure (radial diusivities); White matter microstructure (mean diusivities); Schizophrenia; schizoaffective disorder or bipolar disorder; Pulse pressure; Schizophrenia or bipolar disorder; Lung cancer; Urate levels
rs1811359	*HLA.DPB2*	confirmed	Hepatitis B; Aspirin exacerbated respiratory disease in asthmatics; Hepatitis B (viral clearance); Severe aplastic anemia; Chronic hepatitis B infection; Hepatitis B vaccine response; Wegener's granulomatosis; Antineutrophil cytoplasmic antibody-associated vasculitis; Mouth ulcers; Response to hepatitis B vaccine; Knee osteoarthritis; Osteoarthritis of the hip or knee; Osteoarthritis; White coat effect (clinic diastolic blood pressure minus ambulatory diastolic blood pressure); Thyroid peroxidase antibody levels; Acute graft versus host disease in bone marrow transplantation (recipient effect); Susceptibility to persistent hepatitis B virus infection; EGFR mutation-positive lung adenocarcinoma; SjÃ¶gren's syndrome

eQTL rs ID	Gene symbol	confirmation	GWAS Catalog phenotypes
rs7797295	*GNA12*	confirmed	Height; Ulcerative colitis; Birth weight; Body fat distribution (trunk fat ratio); Body fat distribution (leg fat ratio); Heel bone mineral density; White blood cell count; Chronic obstructive pulmonary disease; Lung function (FEV1/FVC); White matter microstructure (mode of anisotropy); Brain region volumes; Hip circumference; Hip circumference adjusted for BMI; Waist circumference adjusted for body mass index; Waist circumference adjusted for BMI in non-smokers; Waist circumference adjusted for BMI (joint analysis main effects and physical activity interaction); Inflammatory bowel disease; Waist circumference adjusted for BMI in active individuals; Waist circumference adjusted for BMI (adjusted for smoking behaviour); Waist circumference adjusted for BMI (joint analysis main effects and smoking interaction)
rs80233585	*PPP1R16A*	confirmed	General cognitive ability; Reaction time; Lung function (FVC); Cognitive ability; years of educational attainment or schizophrenia (pleiotropy); Height; Blood metabolite levels; Age at first birth; Mean corpuscular hemoglobin concentration; Educational attainment (years of education); Smoking initiation (ever regular vs never regular) (MTAG); Self-reported math ability (MTAG); Self-reported math ability; Highest math class taken; Educational attainment (MTAG); Cognitive performance (MTAG); Highest math class taken (MTAG)
rs1545837	*KIAA1967*	confirmed	Parkinson's disease or first degree relation to individual with Parkinson's disease; Lung function (FVC); Triglycerides; Parkinson's disease; Exploratory eye movement dysfunction in schizophrenia (responsive search score); Exploratory eye movement dysfunction in schizophrenia (cognitive search score)
rs11778927	*RP11.51J9.5*	confirmed	Lung function (FEV1/FVC); Migraine
rs3213849	*FGFR1*	confirmed	Lung function (FEV1/FVC); PR interval; Body mass index; Age of smoking initiation (MTAG); Smoking initiation (ever regular vs never regular) (MTAG); Response to cognitive-behavioural therapy in anxiety disorder; Waist-to-hip ratio adjusted for BMI; Waist-hip ratio
rs10870201	*CARD9*	confirmed	Crohn's disease; Ulcerative colitis; Red cell distribution width; Lung function (FVC); FEV1; Type 2 diabetes; Glucose homeostasis traits; Ankylosing spondylitis; Inflammatory bowel disease; Peak insulin response (insulin secretion adjusted); Pediatric autoimmune diseases; IgA nephropathy; Granulocyte percentage of myeloid white cells; Chronic inflammatory diseases (ankylosing spondylitis; Crohn's disease; psoriasis; primary sclerosing cholangitis; ulcerative colitis) (pleiotropy)
rs10870160	*SDCCAG3*	confirmed	Crohn's disease; Ulcerative colitis; Red cell distribution width; White blood cell count; Lung function (FVC); Morningness; Morning person; Glucose homeostasis traits; Ankylosing spondylitis; Inflammatory bowel disease; Chronotype; Basal cell carcinoma; Squamous cell carcinoma; Cutaneous squamous cell carcinoma; Pediatric autoimmune diseases; IgA nephropathy; Eosinophil counts; Chronic inflammatory diseases (ankylosing spondylitis; Crohn's disease; psoriasis; primary sclerosing cholangitis; ulcerative colitis) (pleiotropy); Myeloid white cell count; Sum neutrophil eosinophil counts; Granulocyte count; Sum basophil neutrophil counts; Neutrophil count

Supplementary Table 4 Significant KEGG pathways from the enrichment analysis of genes with novel eQTL in the PAH Cohort.

Column names: eQTL chr:pos (REF/ALT) = eQTL chromosome: base pair position (reference/alternative) alleles in the Ensembl human genome reference GRCh37; eQTL rs ID = dbSNP (150) variant identifier; eQTL type = variant annotation from ANNOVAR.

**Gene symbol**	**Gene name**	**eQTL chr:pos (REF/ALT)**	**eQTL rs ID**	**cis/trans**	**eQTL type**	**eQTL P-value**
Taste transduction						
TAS2R13	taste 2 receptor member 13	12:11331094 (T/C)	rs7350611	cis	intergenic	$5.99x10^{-20}$
TAS2R19	taste 2 receptor member 19	12:11138683 (C/T)	rs10772397	cis	exonic	$1.43x10^{-16}$
TAS2R30	taste 2 receptor member 30	12:11140444 (T/C)	rs6488334	cis	ncRNA intronic	$9.02x10^{-13}$
TAS2R31	taste 2 receptor member 31	12:10944783 (A/G)	rs2588347	cis	ncRNA intronic	$4.88x10^{-19}$
TAS2R41	taste 2 receptor member 41	7:143081942 (G/C)	rs11772895	cis	ncRNA intronic	$3.57x10^{-37}$
TAS2R43	taste 2 receptor member 43	12:11226237 (A/G)	rs67933574	cis	ncRNA intronic	$8.58x10^{-65}$
TAS2R46	taste 2 receptor member 46	12:11140444 (T/C)	rs6488334	cis	ncRNA intronic	$5.15x10^{-22}$
TAS2R50	taste 2 receptor member 50	12:11138683 (C/T)	rs10772397	cis	exonic	$2.98x10^{-28}$
Graft-versus-host disease						
HLA-B	major histocompatibility complex, class I, B	6:31322782 (C/G)	rs2596496	cis	intronic	$6.12x10^{-23}$
HLA-C	major histocompatibility complex, class I, C	6:31239681 (A/T)	rs9264669	cis	intronic	$4.11x10^{-39}$
HLA-DQA1	major histocompatibility complex, class II, DQ alpha 1	6:32610314 (T/C)	rs9272779	cis	intronic	$2.64x10^{-19}$
HLA-DRB5	major histocompatibility complex, class II, DR beta 5	6:32501347 (G/A)	rs67851010	cis	intergenic	$4.55x10^{-42}$
HLA-G	major histocompatibility complex, class I, G	6:31242089 (A/T)	rs2524080	trans	intergenic	$8.51x10^{-16}$
HLA-G	major histocompatibility complex, class I, G	6:29936065 (C/T)	rs17180100	cis	intergenic	$1.59x10^{-28}$
KIR3DL1	killer cell immunoglobulin like receptor, three Ig domains and cytoplasmic tail 1	19:55338833 (G/A)	rs3117640	cis	intronic	$4.03x10^{-22}$
Autoimmune thyroid disease						
HLA-B	major histocompatibility complex, class I, B	6:31322782 (C/G)	rs2596496	cis	intronic	$6.12x10^{-23}$
HLA-C	major histocompatibility complex, class I, C	6:31239681 (A/T)	rs9264669	cis	intronic	$4.11x10^{-39}$
HLA-DQA1	major histocompatibility complex, class II, DQ alpha 1	6:32610314 (T/C)	rs9272779	cis	intronic	$2.64x10^{-19}$
HLA-DRB5	major histocompatibility complex, class II, DR beta 5	6:32501347 (G/A)	rs67851010	cis	intergenic	$4.55x10^{-42}$
HLA-G	major histocompatibility complex, class I, G	6:31242089 (A/T)	rs2524080	trans	intergenic	$8.51x10^{-16}$
HLA-G	major histocompatibility complex, class I, G	6:29936065 (C/T)	rs17180100	cis	intergenic	$1.59x10^{-28}$
TG	thyroglobulin	8:133791175 (C/G)	rs988067	cis	intronic	$5.79x10^{-25}$
Allograft rejection						
HLA-B	major histocompatibility complex, class I, B	6:31322782 (C/G)	rs2596496	cis	intronic	$6.12x10^{-23}$
HLA-C	major histocompatibility complex, class I, C	6:31239681 (A/T)	rs9264669	cis	intronic	$4.11x10^{-39}$
HLA-DQA1	major histocompatibility complex, class II, DQ alpha 1	6:32610314 (T/C)	rs9272779	cis	intronic	$2.64x10^{-19}$
HLA-DRB5	major histocompatibility complex, class II, DR beta 5	6:32501347 (G/A)	rs67851010	cis	intergenic	$4.55x10^{-42}$
HLA-G	major histocompatibility complex, class I, G	6:31242089 (A/T)	rs2524080	trans	intergenic	$8.51x10^{-16}$
HLA-G	major histocompatibility complex, class I, G	6:29936065 (C/T)	rs17180100	cis	intergenic	$1.59x10^{-28}$
Type I diabetes						
HLA-B	major histocompatibility complex, class I, B	6:31322782 (C/G)	rs2596496	cis	intronic	$6.12x10^{-23}$
HLA-C	major histocompatibility complex, class I, C	6:31239681 (A/T)	rs9264669	cis	intronic	$4.11x10^{-39}$
HLA-DQA1	major histocompatibility complex, class II, DQ alpha 1	6:32610314 (T/C)	rs9272779	cis	intronic	$2.64x10^{-19}$
HLA-DRB5	major histocompatibility complex, class II, DR beta 5	6:32501347 (G/A)	rs67851010	cis	intergenic	$4.55x10^{-42}$

HLA-G	major histocompatibility complex, class I, G	6:31242089 (A/T)	rs2524080	trans	intergenic	$8.51x10^{-16}$
HLA-G	major histocompatibility complex, class I, G	6:29936065 (C/T)	rs17180100	cis	intergenic	$1.59x10^{-28}$

NIHR BioResource Collaborators

NIHR BioResource – Rare Disease Consortium[1], Julian Adlard[2], Munaza Ahmed[3], Tim Aitman[4,5], Hana Alachkar[6], David Allsup[7], Jeff Almeida-King[8], Philip Ancliff[9], Richard Antrobus[10], Ruth Armstrong[11,12,13], Gavin Arno[14,15], Sofie Ashford[1,16], William Astle[1,16,17], Anthony Attwood[1,16], Chris Babbs[18,19], Tamam Bakchoul[20], Tadbir Bariana[21,22], Julian Barwell[23,24], David Bennett[25], David Bentley[26], Agnieszka Bierzynska[27], Tina Biss[28], Marta Bleda[29], Harm Bogaard[30], Christian Bourne[26], Sara Boyce[31], John Bradley[1], Gerome Breen[32,33], Paul Brennan[34,35], Carole Brewer[36], Matthew Brown[1,16], Michael Browning[37], Rachel Buchan[38,39], Matthew Buckland[40], Teofila Bueser[41,42,43], Siobhan Burns[40], Oliver Burren[29], Paul Calleja[44], Gerald Carr-White[42], Keren Carss[1,16], Ruth Casey[11,12,13], Mark Caulfield[45], John Chambers[46,47], Jennifer Chambers[48,49], Floria Cheng[49], Patrick F Chinnery[1,50,51], Martin Christian[52], Colin Church[53], Naomi Clements Brod[1,16], Gerry Coghlan[40], Elizabeth Colby[27], Trevor Cole[54], Janine Collins[55], Peter Collins[56], Camilla Colombo[26], Robin Condliffe[57], Stuart Cook[38,58,59,60], Terry Cook[61], Nichola Cooper[62], Paul Corris[63,64], Abigail Crisp-Hihn[1,16], Nicola Curry[65], Cesare Danesino[66], Matthew Daniels[67,68], Louise Daugherty[1,16], John Davis[1,16], Sri V V Deevi[1,16], Timothy Dent[68], Eleanor Dewhurst[1,16], Peter Dixon[48], Kate Downes[1,16], Anna Drazyk[69], Elizabeth Drewe[70], Tina Dutt[71], David Edgar[72], Karen Edwards[1,16], William Egner[73], Wendy Erber[74], Marie Erwood[1,16], Maria C Estiu[75], Gillian Evans[76], Dafydd Gareth Evans[77], Tamara Everington[78], Mélanie Eyries[79], Remi Favier[80,81,82], Debra Fletcher[1,16], James Fox[1,16], Amy Frary[1,16], Courtney French[83], Kathleen Freson[84], Mattia Frontini[1,16], Daniel Gale[85], Henning Gall[86], Claire Geoghegan[26], Terry Gerighty[26], Stefano Ghio[87], Hossein-Ardeschir Ghofrani[62,86], Simon Gibbs[38], Kimberley Gilmour[88], Barbara Girerd[89,90,91], Sarah Goddard[92], Keith Gomez[21,22], Pavels Gordins[93], David Gosal[6], Stefan Gräf[1,16,29], Luigi Grassi[1,16], Daniel Greene[1,16,17], Lynn Greenhalgh[94], Andreas Greinacher[95], Paolo Gresele[96], Philip Griffiths[97,98], Sofia Grigoriadou[99], Russell Grocock[26], Detelina Grozeva[11], Scott Hackett[100], Charaka Hadinnapola[29], William Hague[101], Matthias Haimel[1,16,29], Matthew Hall[70], Helen Hanson[94], Kirsty Harkness[102], Andrew Harper[38,67,103], Claire Harris[64], Daniel Hart[55], Ahamad Hassan[104], Grant Hayman[105], Alex Henderson[106], Jonathan Hoffmann[54], Rita Horvath[107,108], Arjan Houweling[30], Luke Howard[38], Fengyuan Hu[1,16], Gavin Hudson[107], Joseph Hughes[26], Aarnoud Huissoon[100], Marc Humbert[89,90,91], Sean Humphray[26], Sarah Hunter[26], Matthew Hurles[109], Louise Izatt[110], Roger James[1,16], Sally Johnson[111], Stephen Jolles[112,113], Jennifer Jolley[1,16], Neringa Jurkute[14,22], Mary Kasanicki[114], Hanadi Kazkaz[115], Rashid Kazmi[31], Peter Kelleher[39], David Kiely[57], Nathalie Kingston[1], Robert Klima[44], Myrto Kostadima[1,16], Gabor Kovacs[116,117], Ania Koziell[118,119], Roman Kreuzhuber[1,16], Taco Kuijpers[120,121], Ajith Kumar[3], Dinakantha Kumararatne[114], Manju Kurian[122,123], Michael Laffan[124,125], Fiona Lalloo[77], Michele Lambert[126,127], Hana Lango Allen[1,16], Allan Lawrie[128], Mark Layton[124], Claire Lentaigne[124,125], Adam Levine[85], Rachel Linger[1,16], Hilary Longhurst[99], Eleni Louka[18,19], Robert MacKenzie Ross[129], Bella Madan[130], Eamonn Maher[11,131], Jesmeen Maimaris[88], Sarah Mangles[132], Rutendo Mapeta[1,16], Kevin Marchbank[64], Stephen Marks[9], Hugh S Markus[69], Hanns-Ulrich Marschall[133], Andrew Marshall[134,135,136], Jennifer Martin[1,16,29], Mary Mathias[137], Emma Matthews[22,138], Heather Maxwell[139], Paul McAlinden[64], Mark McCarthy[19,103,140], Stuart Meacham[1,16], Adam Mead[141], Karyn Megy[1,16], Sarju Mehta[142], Michel Michaelides[14], Carolyn Millar[124,125], Shahin Moledina[9], David Montani[89,90,91], Tony Moore[14,15], Nicholas Morrell[1,29], Monika Mozere[85], MPGN/C3 Glomerulopathy Rare Renal Disease group[143], Keith Muir[144], Andrew Mumford[145,146], Michael Newnham[29], Jennifer O'Sullivan[130], Samya Obaji[56], Steven Okoli[18,19], Andrea Olschewski[116], Horst Olschewski[116,117], Kai Ren Ong[54], Elizabeth Ormondroyd[67,68], Willem Ouwehand[1,16], Sofia Papadia[1,16], Soo-Mi Park[12,13,147], David Parry[5], Joan Paterson[11,12,13], Andrew Peacock[53], John Peden[26], Kathelijne Peerlinck[84], Christopher Penkett[1,16], Joanna Pepke-Zaba[148], Romina Petersen[1,16], Angela Pyle[107], Stuart Rankin[44], Anupama Rao[9], F Lucy Raymond[1,11], Paula Rayner-Matthews[1,16], Christine Rees[26], Augusto Rendon[45], Tara Renton[43], Andrew Rice[149,150], Sylvia Richardson[17], Alex Richter[10], Irene Roberts[18,19,151], Catherine Roughley[76], Noemi Roy[18,19,151], Omid Sadeghi-Alavijeh[85], Moin Saleem[27], Nilesh Samani[152], Alba Sanchis-Juan[1,16], Ravishankar Sargur[73], Simon Satchell[27], Sinisa Savic[153], Laura Scelsi[87], Sol Schulman[154], Marie Scully[115], Claire Searle[155], Werner Seeger[86], Carrock Sewell[156], Denis Seyres[1,16], Susie Shapiro[65], Olga Sharmardina[1,16], Rakefet

Shtoyerman[157], Keith Sibson[137], Lucy Side[3], Ilenia Simeoni[1,16], Michael Simpson[158], Suthesh Sivapalaratnam[55], Anne-Bine Skytte[159], Katherine Smith[45], Kenneth G C Smith[29,160], Katie Snape[161], Florent Soubrier[79], Simon Staines[1,16], Emily Staples[29], Hannah Stark[1,16], Jonathan Stephens[1,16], Kathleen Stirrups[1,16], Sophie Stock[1,16], Jay Suntharalingam[129], Emilia Swietlik[29], R Campbell Tait[162], Kate Talks[28], Rhea Tan[69], James Thaventhiran[29], Andreas Themistocleous[25], Moira Thomas[163], Kate Thomson[67,68], Adrian Thrasher[9], Chantal Thys[84], Marc Tischkowitz[164], Catherine Titterton[1,16], Cheng-Hock Toh[71], Mark Toshner[29], Matthew Traylor[69], Carmen Treacy[29,148], Richard Trembath[41], Salih Tuna[1,16], Wojciech Turek[44], Ernest Turro[1,16,17], Tom Vale[25], Chris Van Geet[84], Natalie Van Zuydam[25], Marta Vazquez-Lopez[49], Julie von Ziegenweidt[1,16], Anton Vonk Noordegraaf[30], Quintin Waisfisz[30], Suellen Walker[9], James Ware[38,39,58], Hugh Watkins[67,68,103], Christopher Watt[1,16], Andrew Webster[14,15], Wei Wei[50], Steven Welch[100], Julie Wessels[92], Sarah Westbury[145,146], John-Paul Westwood[115], John Wharton[62], Deborah Whitehorn[1,16], James Whitworth[11,12,13], Martin R Wilkins[62], Catherine Williamson[48,165], Edwin Wong[98], Nicholas Wood[166,167], Yvette Wood[1,16], Geoff Woods[11,114], Emma Woodward[77], Stephen Wort[39,41], Austen Worth[9], Katherine Yates[1,16,29], Patrick Yong[168], Tim Young[1,16], Ping Yu[1,16], Patrick Yu-Wai-Man[50]

Affiliations

[1]NIHR BioResource, Cambridge University Hospitals NHS Foundation, Cambridge Biomedical Campus, Cambridge, UK. [2]Chapel Allerton Hospital, Leeds Teaching Hospitals NHS Trust, Leeds, UK. [3]North East Thames Regional Genetics Service, Great Ormond Street Hospital for Children NHS Foundation Trust, London, UK. [4]MRC Clinical Sciences Centre, Faculty of Medicine, Imperial College London, London, UK. [5]Institute of Genetics and Molecular Medicine, University of Edinburgh, Edinburgh, UK. [6]Salford Royal NHS Foundation Trust, Salford, UK. [7]Queens Centre for Haematology and Oncology, Castle Hill Hospital, Hull and East Yorkshire NHS Trust, Cottingham, UK. [8]European Molecular Biology Laboratory, European Bioinformatics Institute (EMBL-EBI), Wellcome Genome Campus, Hinxton, Cambridge, UK. [9]Great Ormond Street Hospital for Children NHS Foundation Trust, London, UK. [10]University Hospitals Birmingham NHS Foundation Trust, Birmingham, UK. [11]Department of Medical Genetics, Cambridge Institute for Medical Research, University of Cambridge, Cambridge Biomedical Campus, Cambridge, UK. [12]Cancer Research UK Cambridge Centre, Cambridge Biomedical Campus, Cambridge, UK. [13]NIHR Cambridge Biomedical Research Centre, Cambridge Biomedical Campus, Cambridge, UK. [14]Moorfields Eye Hospital NHS Foundation Trust, London, UK. [15]UCL Institute of Ophthalmology, University College London, London, UK. [16]Department of Haematology, University of Cambridge, Cambridge Biomedical Campus, Cambridge, UK. [17]MRC Biostatistics Unit, Cambridge Institute of Public Health, University of Cambridge, Cambridge, UK. [18]MRC Molecular Haematology Unit, Weatherall Institute of Molecular Medicine, University of Oxford, Oxford, UK. [19]NIHR Oxford Biomedical Research Centre, Oxford University Hospitals Trust, Oxford, UK. [20]Center for Clinical Transfusion Medicine, University Hospital of Tübingen, Tübingen, Germany. [21]The Katharine Dormandy Haemophilia Centre and Thrombosis Unit, Royal Free London NHS Foundation Trust, London, UK. [22]University College London, London, UK. [23]Department of Clinical Genetics, Leicester Royal Infirmary, University Hospitals of Leicester, Leicester, UK. [24]University of Leicester, Leicester, UK. [25]The Nuffield Department of Clinical Neurosciences, University of Oxford, John Radcliffe Hospital, Oxford, UK. [26]Illumina Limited, Chesterford Research Park, Little Chesterford, Nr Saffron Walden, UK. [27]Bristol Renal, University of Bristol, Bristol, UK. [28]Haematology Department, Royal Victoria Infirmary, The Newcastle upon Tyne Hospitals NHS Foundation Trust, Newcastle upon Tyne, UK. [29]Department of Medicine, School of Clinical Medicine, University of Cambridge, Cambridge Biomedical Campus, Cambridge, UK. [30]Department of Pulmonary Medicine, VU University Medical Centre, Amsterdam, The Netherlands. [31]Southampton General Hospital, University Hospital Southampton NHS Foundation Trust, Southampton, UK. [32]MRC Social, Genetic & Developmental Psychiatry Centre, Institute of Psychiatry, Psychology & Neuroscience, King's College London,

London, UK. [33]NIHR Biomedical Research Centre for Mental Health, Maudsley Hospital, London, UK. [34]Newcastle University, Newcastle upon Tyne, UK. [35]Newcastle upon Tyne Hospitals NHS Foundation Trust, Newcastle upon Tyne, UK. [36]Department of Clinical Genetics, Royal Devon & Exeter Hospital, Royal Devon and Exeter NHS Foundation Trust, Exeter, UK. [37]Department of Immunology, Leicester Royal Infirmary, Leicester, UK. [38]National Heart and Lung Institute, Imperial College London, Royal Brompton Hospital, London, UK. [39]Royal Brompton Hospital, Royal Brompton and Harefield NHS Foundation Trust, London, UK. [40]Royal Free London NHS Foundation Trust, London, UK. [41]King's College London, London, UK. [42]Guy's and St Thomas' Hospital, Guy's and St Thomas' NHS Foundation Trust, London, UK. [43]King's College Hospital NHS Foundation Trust, London, UK. [44]High Performance Computing Service, University of Cambridge, Cambridge, UK. [45]Genomics England Ltd, London, UK. [46]Epidemiology and Biostatistics, Imperial College London, London, UK. [47]Imperial College Healthcare NHS Trust, London, UK. [48]Division of Women's Health, King's College London, London, UK. [49]Women's Health Research Centre, Surgery and Cancer, Faculty of Medicine, Hammersmith Hospital, Imperial College Healthcare NHS Trust, London, UK. [50]Department of Clinical Neurosciences, School of Clinical Medicine, University of Cambridge, Cambridge Biomedical Campus, Cambridge, UK. [51]Medical Research Council Mitochondrial Biology Unit, Cambridge Biomedical Campus, Cambridge, UK. [52]Children's Renal and Urology Unit, Nottingham Children's Hospital, QMC, Nottingham University Hospitals NHS Trust, Nottingham, UK. [53]Golden Jubilee National Hospital, Glasgow, UK. [54]West Midlands Regional Genetics Service, Birmingham Women's and Children's NHS Foundation Trust, Birmingham, UK. [55]The Royal London Hospital, Barts Health NHS Foundation Trust, London, UK. [56]The Arthur Bloom Haemophilia Centre, University Hospital of Wales, Cardiff, UK. [57]Sheffield Pulmonary Vascular Disease Unit, Royal Hallamshire Hospital NHS Foundation Trust, Sheffield, UK. [58]MRC London Institute of Medical Sciences, Imperial College London, London, UK. [59]National Heart Research Institute Singapore, National Heart Centre Singapore, Singapore, Singapore. [60]Division of Cardiovascular & Metabolic Disorders, Duke-National University of Singapore, Singapore, Singapore. [61]Imperial College Renal and Transplant Centre, Hammersmith Hospital, Imperial College Healthcare NHS Trust, London, UK. [62]Department of Medicine, Imperial College London, London, UK. [63]National Pulmonary Hypertension Service (Newcastle), The Newcastle upon Tyne Hospitals NHS Foundation Trust, Newcastle upon Tyne, UK. [64]Institute of Cellular Medicine, Faculty of Medical Sciences, Newcastle University, Newcastle upon Tyne, UK. [65]Oxford Haemophilia and Thrombosis Centre, The Churchill Hospital, Oxford University Hospitals NHS Trust, Oxford, UK. [66]Department of Molecular Medicine, General Biology, and Medical Genetics Unit, University of Pavia, Pavia, Italy. [67]Department of Cardiovascular Medicine, Radcliffe Department of Medicine, University of Oxford, Oxford, UK. [68]Oxford University Hospitals NHS Foundation Trust, Oxford, UK. [69]Stroke Research Group, Department of Clinical Neurosciences, University of Cambridge, Cambridge Biomedical Campus, Cambridge, UK. [70]Nottingham University Hospitals NHS Trust, Nottingham, UK. [71]The Roald Dahl Haemostasis and Thrombosis Centre, The Royal Liverpool Hospital, Liverpool, UK. [72]Regional Immunology Service, Kelvin Building, Royal Victoria Hospital, Belfast, UK. [73]Sheffield Teaching Hospitals NHS Foundation Trust, Sheffield, UK. [74]Pathology and Laboratory Medicine, University of Western Australia, Crawley, Australia. [75]Ramón Sardá Mother's and Children's Hospital, Buenos Aires, Argentina. [76]Haemophilia Centre, Kent & Canterbury Hospital, East Kent Hospitals University Foundation Trust, Canterbury, UK. [77]Manchester Centre for Genomic Medicine, Saint Mary's Hospital, Manchester, UK. [78]Salisbury District Hospital, Salisbury NHS Foundation Trust, Salisbury, UK. [79]Departement de genetique, Hopital Pitie-Salpetriere, Paris, France. [80]Service d'Hématologie Biologique, Hôpital d'enfants Armand Trousseau, Paris, France, Paris, France. [81]Inserm U1170, Villejuif, France. [82]Assistance Publique-Hôpitaux de Paris, Département d'Hématologie, Hôpital Armand Trousseau, Paris, France. [83]Department of Paediatrics, School of Clinical Medicine, University of Cambridge , Cambridge Biomedical Campus, Cambridge, UK. [84]Department of Cardiovascular Sciences, Center for Molecular and Vascular Biology, University of Leuven, Leuven, Belgium. [85]UCL Centre for Nephrology, University College London, London, UK. [86]University of Giessen and Marburg Lung Center (UGMLC), Giessen, Germany. [87]Division of Cardiology, Fondazione IRCCS Policlinico S. Matteo, Pavia, Italy. [88]UCL Great Ormond

Street Institute of Child Health, London, UK. [89]Universite Paris-Sud, Le Kremlin-Bicêtre, France. [90]Service de Pneumologie, DHU Thorax Innovation, Hôpital Bicêtre, Le Kremlin-Bicêtre, France. [91]INSERM U999, LabEx LERMIT, Centre Chirurgical Marie Lannelongue, Le Plessis Robinson, France. [92]University Hospitals of North Midlands NHS Trust, Stoke-on-Trent, UK. [93]East Yorkshire Regional Adult Immunology and Allergy Unit, Hull Royal Infirmary, Hull & East Yorkshire Hospitals NHS Trust, Hull, UK. [94]Department of Clinical Genetics, Liverpool Women's NHS Foundation, Liverpool, UK. [95]Institute for Immunology and Transfusion Medicine, University of Greifswald, Greifswald, Germany. [96]Section of Internal and Cardiovascular Medicine, University of Perugia, Perugia, Italy. [97]Mitochondrial Research Group, Institute of Genetic Medicine, Newcastle University, Newcastle upon Tyne, UK. [98]Institute of Genetic Medicine, Newcastle University, Newcastle upon Tyne, UK. [99]Barts Health NHS Foundation Trust, London, UK. [100]Birmingham Heartlands Hospital, Heart of England NHS Foundation Trust, Birmingham, UK. [101]ARCH: Australian Research Centre for Health of Women and Babies, Robinson Research Institute, Discipline of Obstetrics and Gynaecology, The University of Adelaide, Women's and Children's Hospital, Adelaide, Australia. [102]Department of Neurology, Sheffield Teaching Hospitals NHS Foundation Trust, Sheffield, UK. [103]Wellcome Trust Centre for Human Genetics, University of Oxford, Oxford, UK. [104]Department of Neurology, Leeds Teaching Hospital NHS Trust, Leeds, UK. [105]Epsom & St Helier University Hospitals NHS Trust, London, UK. [106]Northern Genetics Service, The Newcastle upon Tyne Hospitals NHS Foundation Trust, International Centre for Life, Newcastle upon Tyne, UK. [107]Wellcome Centre for Mitochondrial Research, Institute of Genetic Medicine, Newcastle University, Newcastle upon Tyne, UK. [108]John Walton Muscular Dystrophy Research Centre, Institute of Genetic Medicine, Newcastle University, Newcastle upon Tyne, UK. [109]Wellcome Trust Sanger Institute, Hinxton, Cambridge, UK. [110]Department of Clinical Genetics, Guy's and St Thomas' NHS Foundation Trust, London, UK. [111]Department of Paediatric Nephrology, Great North Children's Hospital, Newcastle upon Tyne Hospitals NHS Foundation Trust, Newcastle upon Tyne, UK. [112]University Hospital of Wales, Cardiff, UK. [113]Cardiff & Vale University LHB, Cardiff, UK. [114]Addenbrookes Hospital, Cambridge University Hospitals NHS Foundation Trust, Cambridge, UK. [115]University College London Hospitals NHS Foundation Trust, London, UK. [116]Ludwig Boltzmann Institute for Lung Vascular Research, Graz, Austria. [117]Dept of Internal Medicine, Division of Pulmonology, Medical University of Graz, Graz, Austria. [118]Division of Transplantation Immunology and Mucosal Biology, Department of Experimental Immunobiology, Faculty of Life Sciences and Medicine, King's College London, London, UK. [119]Department of Paediatric Nephrology, Evelina London Children's Hospital, Guy's & St Thomas' NHS Foundation Trust, London, UK. [120]Department of Pediatric Hematology, Immunology, Rheumatology and Infectious Diseases, Emma Children's Hospital, Academic Medical Center (AMC), University of Amsterdam, Amsterdam, The Netherlands. [121]Department of Clinical Genetics, Academic Medical Center (AMC), University of Amsterdam, Amsterdam, The Netherlands. [122]Molecular Neurosciences, Developmental Neurosciences, UCL Great Ormond Street Institute of Child Health, London, UK. [123]Department of Neurology, Great Ormond Street Hospital for Children NHS Foundation Trust, London, UK. [124]Department of Haematology, Hammersmith Hospital, Imperial College Healthcare NHS Trust, London, UK. [125]Department of Haematology, Imperial College London, London, UK. [126]Division of Hematology, The Children's Hospital of Philadelphia, Philadelphia, USA. [127]Department of Pediatrics, Perelman School of Medicine at the University of Pennsylvania, Philadelphia, USA. [128]Department of Infection, Immunity & Cardiovascular Disease, University of Sheffield, Sheffield, UK. [129]Royal United Hospitals Bath NHS Foundation Trust, Bath, UK. [130]Department of Haematology, Guy's and St Thomas' NHS Foundation Trust, London, UK. [131]Cambridge NIHR Biomedical Research Centre, Cambridge Biomedical Campus, Cambridge, UK. [132]Haemophilia, Haemostasis and Thrombosis Centre, Hampshire Hospitals NHS Foundation Trust, Basingstoke, UK. [133]Wallenberg Laboratory, Department of Molecular and Clinical Medicine, Sahlgrenska Academy, University of Gothenburg, Gothenburg, Sweden. [134]Faculty of Medical and Human Sciences, Centre for Endocrinology and Diabetes, Institute of Human Development, University of Manchester, Manchester, UK. [135]Department of Clinical Neurophysiology, Manchester Royal Infirmary, Central Manchester University Hospitals National Health Service Foundation Trust, Manchester Academic Health

Science Centre, Manchester, UK. [136]National Institute for Health Research/Wellcome Trust Clinical Research Facility, Manchester, UK. [137]Department of Haematology, Great Ormond Street Hospital for Children NHS Foundation Trust, London, UK. [138]The National Hospital for Neurology and Neurosurgery, University College London Hospitals NHS Foundation Trust, London, UK. [139]Royal Hospital for Children, NHS Greater Glasgow and Clyde, Glasgow, UK. [140]Oxford Centre for Diabetes, Endocrinology and Metabolism, University of Oxford, Churchill Hospital, Oxford, UK. [141]Centre for Haematology, Department of Medicine, Hammersmith Hospital, Imperial College Healthcare NHS Trust, London, UK. [142]Department of Clinical Genetics, Addenbrookes Hospital, Cambridge University Hospitals NHS Foundation Trust, Cambridge, UK. [143]MPGN/C3 Glomerulopathy Rare Renal Disease group, , UK. [144]Institute of Neuroscience and Psychology, University of Glasgow, Glasgow, UK. [145]School of Cellular and Molecular Medicine, University of Bristol, Bristol, UK. [146]University Hospitals Bristol NHS Foundation Trust, Bristol, UK. [147]East Anglian Regional Genetics Service, Cambridge University Hospitals NHS Foundation Trust, Cambridge, UK. [148]Royal Papworth Hospital NHS Foundation Trust, Cambridge, UK. [149]Pain Research, Department of Surgery and Cancer, Faculty of Medicine, Imperial College London, London, UK. [150]Chelsea and Westminster Hospital NHS Foundation Trust, London, UK. [151]Department of Paediatrics, Weatherall Institute of Molecular Medicine, University of Oxford, Oxford, UK. [152]Departments of Cardiovascular Sciences and NIHR Leicester Cardiovascular Biomedical Research Unit, University of Leicester, Leicester, UK. [153]The Leeds Teaching Hospitals NHS Trust, Leeds, UK. [154]Beth Israel Deaconess Medical Centre and Harvard Medical School, Boston, USA. [155]Department of Clinical Genetics, Nottingham University Hospitals NHS Trust, Nottingham, UK. [156]Scunthorpe General Hospital, Northern Lincolnshire and Goole NHS Foundation Trust, Scunthorpe, UK. [157]Clinical Genetics Institute, Kaplan Medical Center, Rehovot, Israel. [158]Genetics and Molecular Medicine, King's College London, London, UK. [159]Aarhus University Hospital, Aarhus, Denmark. [160]Cambridge Institute for Medical Research, University of Cambridge, Cambridge Biomedical Campus, Cambridge, UK. [161]Department of Clinical Genetics, St George's University Hospitals NHS Foundation Trust, London, UK. [162]Glasgow Royal Infirmary, NHS Greater Glasgow and Clyde, Glasgow, UK. [163]Gartnavel General Hospital, NHS Greater Glasgow and Clyde, Glasgow, UK. [164]Addenbrooke's Treatment Centre, Addenbrooke's Hospital, Cambridge University Hospitals NHS Foundation Trust, Cambridge, UK. [165]Institute of Reproductive and Developmental Biology, Surgery and Cancer, Hammersmith Hospital, Imperial College Healthcare NHS Trust, London, UK. [166]Department of Molecular Neuroscience, UCL Institute of Neurology, London, UK. [167]UCL Genetics Institute, London, UK. [168]Frimley Park Hospital, NHS Frimley Health Foundation Trust, Camberley, UK.
